# Salicylic acid-induced transcriptional reprogramming by the HAC–NPR1–TGA histone acetyltransferase complex in *Arabidopsis*

**DOI:** 10.1093/nar/gky847

**Published:** 2018-09-17

**Authors:** Hongshi Jin, Sun-Mee Choi, Min-Jeong Kang, Se-Hun Yun, Dong-Jin Kwon, Yoo-Sun Noh, Bosl Noh

**Affiliations:** 1 School of Biological Sciences, Seoul National University, Seoul 08826, Korea; 2 Plant Genomics and Breeding Institute, Seoul National University, Seoul 08826, Korea; 3 Research Institute of Basic Sciences, Seoul National University, Seoul 08826, Korea

## Abstract

Plant immunity depends on massive expression of *pathogenesis-related* genes (*PRs*) whose transcription is de-repressed by pathogen-induced signals. Salicylic acid (SA) acts as a major signaling molecule in plant immunity and systemic acquired resistance triggered by bacterial or viral pathogens. SA signal results in the activation of the master immune regulator, Nonexpressor of pathogenesis-related genes 1 (NPR1), which is thought to be recruited by transcription factors such as TGAs to numerous downstream *PRs*. Despite its key role in SA-triggered immunity, the biochemical nature of the transcriptional coactivator function of NPR1 and the massive transcriptional reprogramming induced by it remain obscure. Here we demonstrate that the CBP/p300-family histone acetyltransferases, HACs and NPR1 are both essential to develop SA-triggered immunity and *PR* induction. Indeed HACs and NPR1 form a coactivator complex and are recruited to *PR* chromatin through TGAs upon SA signal, and finally the HAC−NPR1−TGA complex activates *PR* transcription by histone acetylation-mediated epigenetic reprogramming. Thus, our study reveals a molecular mechanism of NPR1-mediated transcriptional reprogramming and a key epigenetic aspect of the central immune system in plants.

## INTRODUCTION

Although plants lack specialized immune cells, they have developed sophisticated defense systems against pathogenic attacks. Salicylic acid (SA), a key signaling molecule in plant immunity (1,2), induces a transcription reprogramming through the master immune-regulator Nonexpressor of pathogenesis-related genes 1 (NPR1) (3–6). NPR1 acts as a transcriptional coactivator for nearly 2000 genes by interacting with transcription factors such as TGACG-BINDING FACTORs (TGAs) (7–11). SA regulates NPR1 activity at multiple levels: (i) SA-triggered redox changes result in NPR1 monomerization and nuclear translocation (12,13); (ii) SA-mediated post-translational modifications of NPR1 influence its transcriptional activity and turnover (14–16); (iii) SA binding to NPR1 causes a conformational change, enabling its transcriptional coactivator function (17–20).

CBP/p300-family histone acetyltransferases (HATs) are well-known transcriptional coactivators that control a variety of differentiation and developmental processes. They facilitate transcription by diverse functions: relaxing chromatin structure through histone acetylation (21–23), modulating the activity of transcriptional regulators through acetylation (24–26), acting as adaptor for numerous transcription factors (27,28) and bridging transcription factors to transcription machineries (29,30). *Arabidopsis* has five *CBP/p300*-family genes: *HAC1, HAC2, HAC4, HAC5* and *HAC12* (31,32). Although multiple morphological and developmental defects were observed in *Arabidopsis* mutants lacking multiple *HACs*, so far only a few studies have reported in-depth physiological analyses on these mutants and revealed the molecular functions of HACs in flowering and ethylene signaling (31,33).

Despite the essential role of NPR1 in SA-triggered transcription of *pathogenesis-related* genes (*PRs*) during plant defense, the molecular mechanism of its transcriptional coactivator role remains elusive. In this study, we show that the CBP/p300-family HATs, HAC1 and HAC5 (HAC1/5), are essential to develop SA-triggered immunity and *PR* induction. HAC1/5 and NPR1 form a coactivator complex and are recruited to *PR* chromatin through NPR1–TGA interaction, finally relaxing repressive local chromatin and facilitating transcription. In sum, our study demonstrates a mechanism of NPR1-mediated transcriptional activation and proposes epigenetic reprogramming as central part of plant immune system.

## MATERIALS AND METHODS

### Plant materials and growth conditions

All the *Arabidopsis* mutants (*hac1-1, hac1-2 hac5-2, hac1-1 hac12-1, haf1-2 haf2-5, hag1-6, ham1-1 ham2-1, npr1-1, tga2 tga5 tga6*) and transgenic plants used in this study are in the Columbia-0 (Col) background. Details of the HAT mutants (34), *npr1-1* (3), and *tga2 tga5 tga6* (Supplementary Table S1) are described elsewhere. List of all the transgenic or multiple-mutant plants used in this study and the ways to generate them are summarized in Supplementary Table S1. All plants were grown under 100 μE m^−2^ s^−1^ cool white fluorescence light under short-day (8-h light/16-h dark photoperiod) or day-neutral (12-h light/12-h dark photoperiod) condition at 22°C. For 2,6-dichloroisonicotinic acid (INA) treatment, 4-week (w)-old plants were sprayed with DW or 300 μM INA (Sigma-Aldrich 456543) as previously described (35). More details of each experimental condition are described in the figure legends.

### Pathogen infection

Pathogen inoculation was performed as described (24). Three days (d) after inoculation, three inoculated leaf discs each from different plants were combined and homogenized in sterile H_2_O, with at least three times of replication. Leaf extracts were plated on King’s B medium and incubated at 28°C for 2 d, and then bacterial growth was determined by counting the colony-forming units.

### Plasmid construction

An *HAC1* genomic DNA including the *HAC1* ORF was generated by polymerase chain reaction (PCR) with HAC1-gate-F/HAC1-R7 (Supplementary Table S2), cloned into pENTR/SD/D-TOPO (Invitrogen K242020), and then recombined into pGWB511, resulting in *35S::HAC1:FLAG-DES*. For the construction of *pNPR1::NPR1:GFP-DES*, an *NPR1* cDNA amplified by PCR with NPR1 ORF-F (NdeI)/NPR1 ORF-R (Supplementary Table S2) was cloned into pENTR/SD/D-TOPO, and then an *NPR1* promoter covering 1.7 kb upstream of the start codon generated by PCR with NPR1 P-F (NotI)/NPR1 P-R (NdeI) (Supplementary Table S2) was inserted into pENTR/SD/D-TOPO in front of the *NPR1* ORF. Finally, the resulting *pNPR1::NPR1-ENTR* was integrated into pEarlyGate301-GFP in which the HA tag of pEarleyGate301 (36) was replaced by the GFP:6xHis tag from pEarleyGate103 (36). For the construction of *pTGA2::TGA2:FLAG-DES*, a *TGA2* cDNA generated by PCR with TGA2 ORF-F (NdeI)/TGA2-R (w/o stop) (Supplementary Table S2) was cloned into pENTR/SD/D-TOPO and then a *TGA2* promoter covering 1.5 kb upstream of the start codon generated by PCR with TGA2 P-F (NotI)/TGA2 P-R (NdeI) (Supplementary Table S2) was inserted into pENTR/SD/D-TOPO in front of the *TGA2* ORF. Subsequently, *pTGA2::TGA2:FLAG-ENTR* was integrated into ImpGWB510 (37), resulting in *pTGA2::TGA2:FLAG-DES*. All the constructs were introduced into plants by floral dip method (38) via *Agrobacterium tumefaciens* strain GV3101 or C58C1.

### Protein purification and immunoblotting

Proteins from nuclear and non-nuclear fractions were prepared as previously described (39,40). Proteins were quantified using the Protein Assay Kit (Bio-Rad 5000006), separated on sodium dodecyl sulfate polyacrylamide gel electrophoresis (SDS-PAGE), and subsequently transferred onto nitrocellulose membranes (Millipore HATF00010). For the detection of proteins, the following antibodies were used with indicated dilutions: α-HA (1:3000; Abcam ab9110), α-GFP (1:4000; Roche 11814460001), α-TGA2/5 antiserum (1:3000; gift from C. Gatz) (41), α-FLAG (1:3000; Sigma-Aldrich A8592), α-H3 (1:10 000; Abcam ab1791) and α-Tubulin (1:3000; Sigma-Aldrich T9026). Quantification of the signal intensity on immunoblot was performed by using ImageJ (42).

### Co-IP assay

Co-immunoprecipitation (co-IP) assay was performed as previously described (12) with minor modifications. Briefly, total proteins were extracted from 4-w-old plants by grinding in liquid N_2_ and homogenizing in extraction buffer (50 mM Tris–HCl, pH 7.5, 150 mM NaCl, 10 mM MgCl_2_, 5 mM ethylenediaminetetraacetic acid (EDTA), 10% glycerol, 60 μM MG132, 100 mM β**-**glycerophosphate, 20 mM sodium fluoride, protease inhibitors and phenylmethylsulfonyl fluoride (PMSF)). After preclearing with protein-A agarose beads, proteins were incubated with α-HA agarose beads (Sigma-Aldrich A2095) or α-GFP (Roche 11814460001) coupled to protein-A agarose (Santa Cruz sc-2001) at 4°C for 3 h. For protein elution, the beads were boiled in 2× SDS sample buffer, and the supernatant obtained after centrifugation was saved and used for protein detection.

### Yeast-two-hybrid assay

*NPR1* cDNA fragments encoding NPR1 full length, NPR1 BTB/POZ-ANK (1-369 aa), NPR1 Δ370 (370-593 aa) and NPR1 Δ513 (513-593 aa) were amplified by PCR with primers NdeI-NPR1-F/BamHI-NPR1-R, NdeI-NPR1-F/BamHI-NPR1-ANK-R, NdeI-NPR1 Δ370-F/NPR1-Stop-R and NdeI-NPR1 Δ513-F/NPR1-Stop-R, respectively (Supplementary Table S3). *HAC1* cDNA fragments encoding HAC1 full length, HAC1-N (7-896 aa), HAC1-C1 (875-1335 aa), HAC1-C2 (991-1536 aa), HAC1-C3 (1356-1697 aa), TAZ^N^ (624-716 aa) and TAZ^C^ (1575-1667 aa) were generated by PCR with primers NdeI-HAC1-F/SalI-HAC1-Stop-R, SmaI-HAC1-N-F/SalI-HAC1-N-R, NcoI-HAC1-C1-F/BamHI-HAC1-C1-R, NcoI-HAC1-C2-F/BamHI-HAC1-C2-R, NdeI-TAZ^N^-F/TAZ^N^–R and NdeI-TAZ^C^-F/TAZ^C^-R, respectively (Supplementary Table S3). *NPR1* and *HAC1* cDNA fragments were cloned into pGADT7 (Clontech 630442) and pGBKT7 (Clontech 630443) vectors, respectively, and introduced into yeast strain AH109 by lithium acetate method as described in the Clontech yeast protocol handbook. Interactions were assessed by yeast growth on synthetic drop-out medium lacking leucine, tryptophan, adenine and histidine in the presence of 1 or 3 mM 3-AT. Protein extraction from yeast was carried out as described previously (43). Briefly, cells were suspended in lysis buffer (50 mM Tris–HCl, pH 7.5, 150 mM NaCl, 0.01% NP-40, 1 mM EDTA, 100 mM PMSF, 1 mM benzamidine, 1 μg/ml leupeptin and 1 μg/ml pepstatin) followed by bead-beating. Cell extracts were centrifuged at 1600 g for 10 min at 4°C and the supernatant was subjected to SDS-PAGE. BD-fusion proteins were detected by using anti-Myc antibody (Merck 05-724) at 1:1500 dilution, whereas AD-fusion proteins were detected by using anti-HA antibody (Roche 11867423001) at 1:1500 dilution.

### Gel filtration assay

Proteins were prepared by homogenizing 4-w-old plant tissues in extraction buffer (20 mM Tris–HCl, pH 7.5, 200 mM NaCl, 10% glycerol, 60 μM MG132, 100 mM β**-**glycerophosphate, 20 mM sodium fluoride, protease inhibitors and PMSF) followed by 20 min of incubation at 4°C. After centrifugation at 13 000 rpm for 5 min, the supernatant was saved and filtered through a 0.45 μm filter (Millipore SLHP033RS). About 1.5 mg of total proteins were injected on the Superdex 200 10/300GL column (GE Healthcare Life Sciences GE17-5175-01) and fractionated by the AKTA fast protein liquid chromatography system (Amersham Biosciences). Proteins in each fraction were concentrated using acetone, separated by SDS-PAGE, and transferred onto nitrocellulose membranes (Millipore HATF00010) for immunoblot analysis.

### RNA extraction and RT-qPCR analysis

Total RNA extraction and reverse transcription were performed as described previously (35). The sequences of primers used for reverse transcription followed by quantitative real-time PCR (RT-qPCR) are provided in Supplementary Table S4.

### ChIP assay

Chromatin immunoprecipitation (ChIP) assay was performed as previously described (31,35). Antibodies used for ChIP were α-H3Ac (histone H3 acetylation) (Millipore 06-599), α-FLAG (Sigma-Aldrich F1804), α-HA (Abcam ab9110) and α-GFP (Life Technologies A6455). The amount of immunoprecipitated chromatin was measured by qPCR using primers listed in Supplementary Table S5. The 2^−ΔΔ*C*^_T_ method (44) was used to calculate the relative amount of amplified products in samples.

### RNA sequencing analysis

Total RNA was isolated from leaves of 4-w-old short day-grown plants treated with DW or INA for 12 h using Tri Reagent (MRC TR118) and further purified with RNeasy MiniKit (QIAGEN 74106) to obtain OD_260/280_ ratio of 1.8 to 2.2. RNAs obtained from three biologically independent experiments were combined and used for RNA-seq preparation. RNA-seq library was constructed and sequenced on the Illumina HiSeq^™^ 2000 at Beijing Genomics Institute (Hong Kong). Reads were aligned to the *Arabidopsis* reference genome using SOAPaligner/soap2 allowing mismatches of no more than 2 bases. Gene-expression level was calculated by using RPKM (reads per kb per million reads) method. Differentially expressed genes (DEGs) were selected with False discovery rate (FDR) ≤ 0.01 and |log_2_ Ratio| ≥ 1 as thresholds.

### ChIP sequencing analysis

ChIP was performed as previously described (31,35) with minor modifications. Protein–DNA immune-complex was precipitated using agarose A beads (Santa Cruz sc-2001) instead of salmon sperm DNA/Protein A agarose beads to avoid the contamination of ChIPed DNA with salmon sperm DNA. About 12–20 ng of DNA pooled from six independent ChIPs was used for library construction after quality check with 2100 Bioanalyzer (Agilent). Library construction and sequencing on Illumina HiSeq^™^ 2000 were performed at Beijing Genomics Institute (Hong Kong). Reads were aligned to the TAIR10 Arabidopsis genome by using SOAP2 aligner and BWA, and uniquely mapped reads were used for further analysis. Using MACS2 version 2.1.0, normalized signals respective to Col input were obtained, and H3Ac-enriched peaks were identified (*P* < 1.00e-02). The wiggle files obtained from peak scanning were visualized and analyzed by using Integrative Genomics Viewer (IGV). Differential peaks between genotypes and/or treatments were identified by using MACS2 bdgdiff (45) (log_10_ likelihood ratio = 1) and annotated by PAVIS (46) (https://manticore.niehs.nih.gov/pavis2/). H3Ac-distribution analysis was performed by using computeMatrix and plotprofile installed in the public server at the Galaxy (https://usegalaxy.org/) (47).

### Sequential ChIP assay

Sequential ChIP was performed as previously described (48) with minor modifications. Chromatin was isolated from cross-linked samples by using 450 ml of nuclei lysis buffer (50 mM Tris–HCl, pH 8.0, 10 mM EDTA, 1% SDS, 0.1 mM PMSF and protease inhibitors), fragmented by sonication and subjected to immunoprecipitation with anti-HA antibody (Abcam ab9110). Immune complexes were eluted by gentle agitation in 100 μl of elution buffer (16.7 mM Tris–HCl, pH 8.0, 1.2 mM EDTA, 20 mM DTT and 1% SDS) at 37°C for 30 min. Eluted chromatin was diluted with 20-fold of ChIP dilution buffer (16.7 mM Tris–HCl, pH 8.0, 1.2 mM EDTA, 167 mM NaCl and 1.1% Triton X-100), subjected to the second immunoprecipitation with anti-GFP (Roche 11814460001) or control anti-FLAG (Sigma-Aldrich A8592) antibody and then eluted with elution buffer (1% SDS and 0.1 M NaHCO_3_). DNA was isolated by reverse-crosslinking and proteinase K treatment and purified using QIAquick PCR Purification Kit (QIAGEN 28106). Quantification of immunoprecipitated DNA and the evaluation of the relative amount of amplified products in samples were performed as described in the ChIP assay section.

## RESULTS

### CBP/p300-family histone acetyltransferases (HACs) activate SA-dependent plant immunity by promoting *PR* transcription through histone acetylation

**Figure 1.**
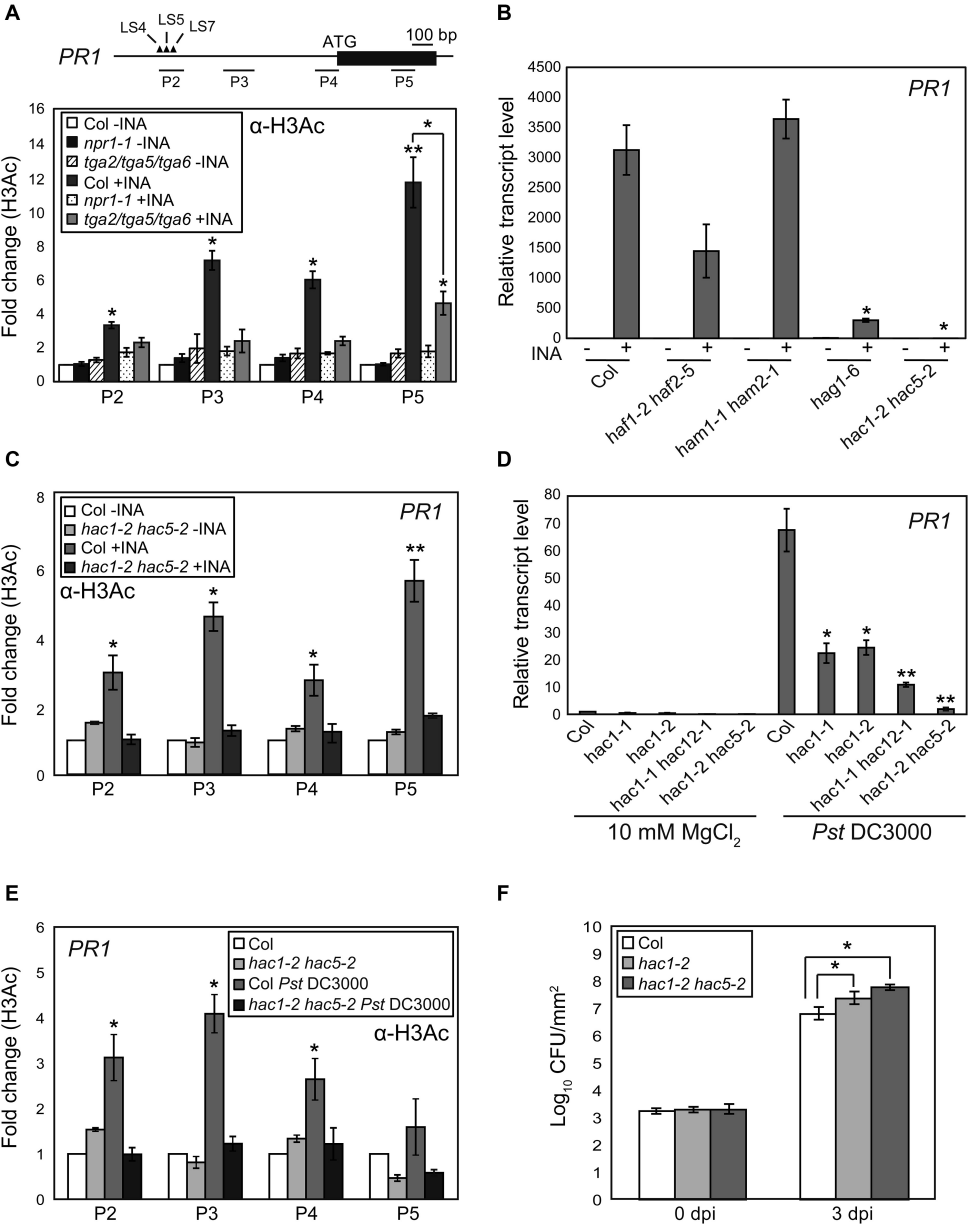
HAC1 and HAC5 are essential for *PR1* transcription and plant immunity. (**A**) H3Ac levels within *PR1* chromatin in Col, *npr1* and *tga2/5/6*. Schematics of *PR1* showing regions tested for ChIP-qPCR. Black box, exon; solid lines, upstream or downstream regions. Closed triangles represent *cis*-elements in the *PR1* promoter: LS4/LS5 and LS7 were proposed to act as negative and positive elements, respectively (51). (**B**) INA-induced *PR1* expression in Col and various HAT mutants. (**C**) H3Ac levels within *PR1* chromatin in Col and *hac1/5*. (**D**) *PR1* expression in Col and *hac* mutants after *Pst* DC3000 infection. (**E**) H3Ac levels within *PR1* chromatin in Col and *hac1/5* after *Pst* DC3000 infection. (**F**) Bacterial cell growth at 0 and 3 d post-infection (dpi) shown as the means ±SE of colony-forming units (CFU) from three biological replicates. Values are the means ±SE of three biological experiments performed in triplicates (A–E). For ChIP-qPCR analyses (A, C and E), untreated WT levels were set to 1 after normalization by input and the internal control *ACTIN2*. For RT-qPCR analyses (B and D), values were normalized to *UBQ10*. Asterisks indicate statistically significant differences compared to Col-INA (A and C), Col+INA (A and B), infected Col (D and F) or uninfected Col (E) (*P < 0.05 and **P < 0.01 in a Student’s *t*-test). All plants were grown on soil for 4 w under day-neutral condition (12-h light/12-h dark photoperiod) and treated with distilled water (DW; -INA) or INA (+INA) for 24 h before harvest (A–C). Pathogen-treated plant samples were harvested at 48 h after infection (D and E).

We and others have found that histone H3 acetylation (H3Ac) at the *Arabidopsis PR1* locus is increased by pathogen attack or SA treatment, and this increase is tightly associated with *PR1* transcription (35,49–51). Interestingly, the H3Ac increase at *PR1* is undermined by the loss of either NPR1 or the three related Class II TGAs (TGA2, TGA5 and TGA6; TGA2/5/6) (50) (Figure 1A). These inspired us to identify HATs responsible for the SA-induced H3Ac (Supplementary Figure S1A). As H3Ac acts as an active epigenetic mark, first we searched for *Arabidopsis* HAT mutants with impaired *PR1* and *PR2* induction upon 2,6-dichloroisonicotinic acid (INA; synthetic SA analog) treatment. The mutants lacking HAG1 (*hag1-6*) (52) or HAC1 and HAC5 (*hac1-2 hac5-2*; *hac1/5*) (31) showed severely impaired INA-induced *PR1* and *PR2* transcriptions (Figure 1B and Supplementary Figure S1B). Further, the INA-induced H3Ac increase at *PR1* was barely detectable in *hac1/5* (Figure 1C) as in *npr1* and *tga2/5/6*, whereas in *hag1-*6 it was comparable to wild-type (WT) in the promoter regions but reduced in the gene body (Supplementary Figure S1C), suggesting that HACs are likely to be the responsible HATs.

**Figure 2.**
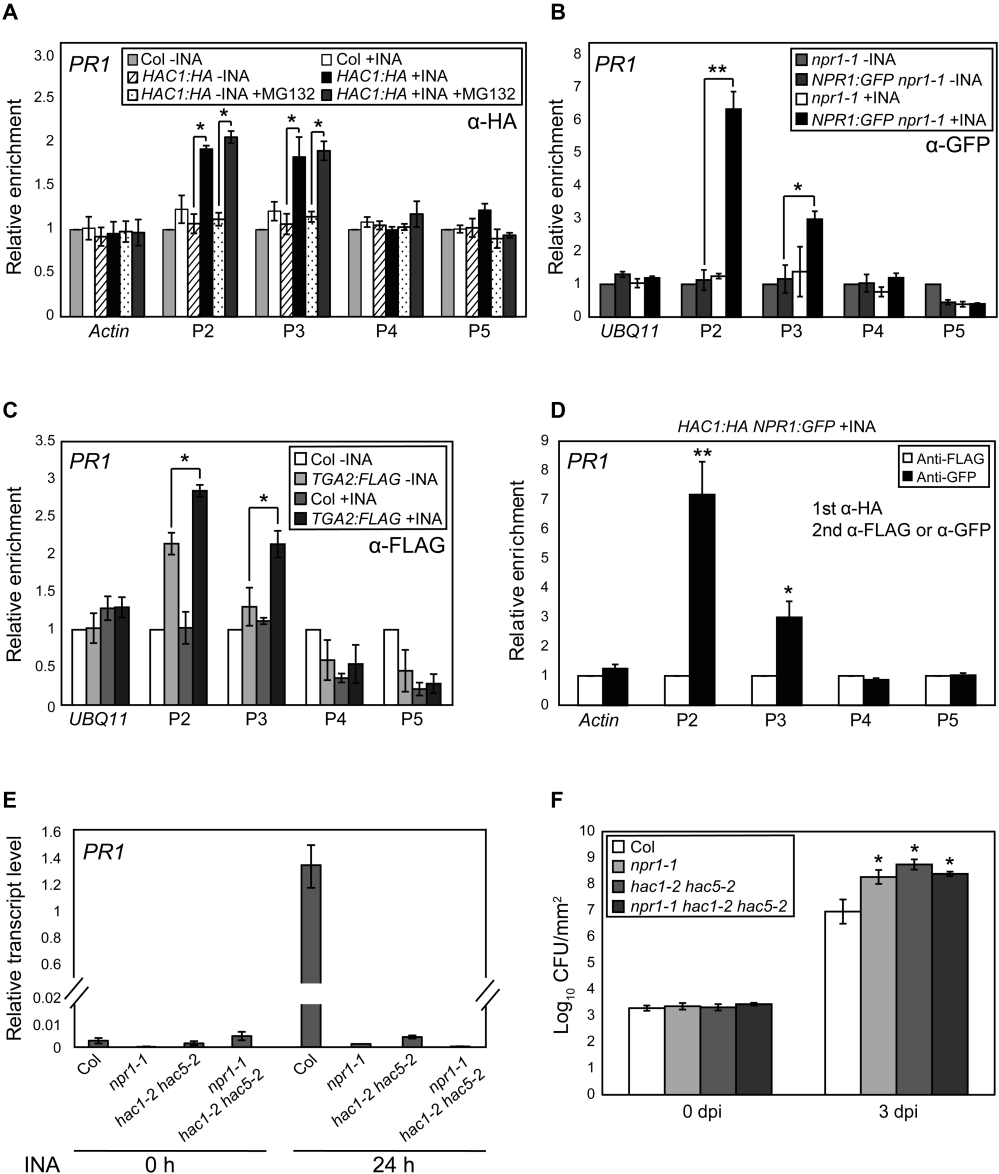
INA signal induces concurrent targeting of HAC1 and NPR1 to *PR1*. (**A**–**C**) INA-induced association of HAC1 (**A**), NPR1 (**B**) and TGA2 (**C**) with *PR1* chromatin as determined by ChIP-qPCR. Level of untagged and untreated Col (A and C) or *npr1* (**B**) was set to 1 after normalization by input. 20 μM of MG132 was (+MG132) or was not added into 1% formaldehyde solution for cross-link before chromatin extraction. (**D**) Co-occupancy of HAC1 and NPR1 at *PR1* loci. Anti-HA immunoprecipitate from INA-treated *HAC1:HA NPR1:GFP* plants was re-immunoprecipitated with anti-FLAG or anti-GFP antibody. The levels of anti-FLAG immunoprecipitates were set to 1 after normalization by input. (**E**) RT-qPCR analysis of *PR1* expression in Col, *npr1, hac1/5* and *npr1 hac1/5* upon INA treatment. Values were normalized to *UBQ10*. (**F**) Bacterial cell growth in Col, *npr1, hac1/5* and *npr1 hac1/5*. The growth of *Pst* DC3000 at 0 or 3 dpi is shown as the means ±SE of CFU from three biological replicates. Asterisks indicate statistically significant differences compared to *HAC1:HA*-INA or *HAC1:HA*-INA+MG132 (A), *NPR1:GFP npr1-1*-INA (B), *TGA2:FLAG*-INA (C), anti-FLAG (D) or infected Col (F) (*P < 0.05 and **P < 0.01 in a Student’s *t*-test). Plants were grown on soil for 4 w under day-neutral (12-h light/12-h dark photoperiod) (A and E) or short-day (8-h light/16-h dark photoperiod) (B–D) condition and treated with DW or INA for 24 h (A and E) or 12 h (B–D) before harvest.

Consistent with the above results, upon infection of *Pseudomonas syringae* pv. tomato DC3000 (*Pst* DC3000), the *PR1/2* induction and H3Ac increase were all severely impaired in *hac1/5* and to lesser extents in *hac1* and *hac1/12* (Figure 1D and E; Supplementary Figure S1D and E). Basal resistance to *Pst* DC3000 was also substantially decreased by the *hac1/5* mutations (Figure 1F). Moreover, a HAC1:HA fusion protein was targeted to the *PR1/2* promoters in both INA- and pathogen-dependent manners (Figure 2A; Supplementary Figures S2A,B and S3), supporting the idea that HACs activate SA-dependent plant immunity by promoting *PR* transcription through histone acetylation.

### HACs form a complex with NPR1 and TGAs and the HAC–NPR1–TGA complex directly induces pathogen- or SA-triggered *PR1/2* transcription

We then determined whether HACs cooperate with NPR1 and TGAs on *PR* chromatin. NPR1:GFP was also targeted to the same *PR1/2*-promoter regions with HAC1:HA in both INA- and pathogen-dependent manners (Figure 2B; Supplementary Figures S2C, S3 and S4). TGA2:FLAG, which can form an INA-induced complex with NPR1:GFP *in vivo* (Supplementary Figure S5), bound independent of INA to the P2 region of the *PR1* promoter (Figure 2C) and the P1 region of the *PR2* promoter (Supplementary Figure S2D), consistent with its reported dual roles as repressor and activator depending on SA signal (7,10,53,54). Interestingly, INA treatment further increased the TGA2:FLAG targeting to these regions and induced a significant targeting to the P3 region of the *PR1* promoter also, resulting in the targeting pattern of TGA2 similar to those of NPR1 and HAC1. This result suggests that INA might induce changes in the biochemical property of TGAs that affect the binding affinity of TGAs to the *PR1/2* promoters or to the antibody used. Sequential ChIP assays using HAC1:HA- and NPR1:GFP-containing transgenic plants showed the presence of *PR1* promoter-bound NPR1:GFP within the HAC1:HA immunoprecipitate (Figure 2D), indicating the co-localization of HAC1 and NPR1 on *PR1* upon INA treatment. These findings, together with the well-known NPR1–TGA interaction and the lack of INA-induced H3Ac increase in *npr1* and *tga2/5/6* mutants (Figure 1A), led us to hypothesize that HACs, NPR1 and TGAs might form a complex on *PR* promoters and modulate transcription through chromatin modification. In support of this view, *hac1/5, npr1* and *npr1 hac1/5* mutants (Supplementary Figure S6) showed comparable INA-induced *PR1* transcript levels and susceptibilities to *Pst* DC3000 (Figure 2E and F).

**Figure 3.**
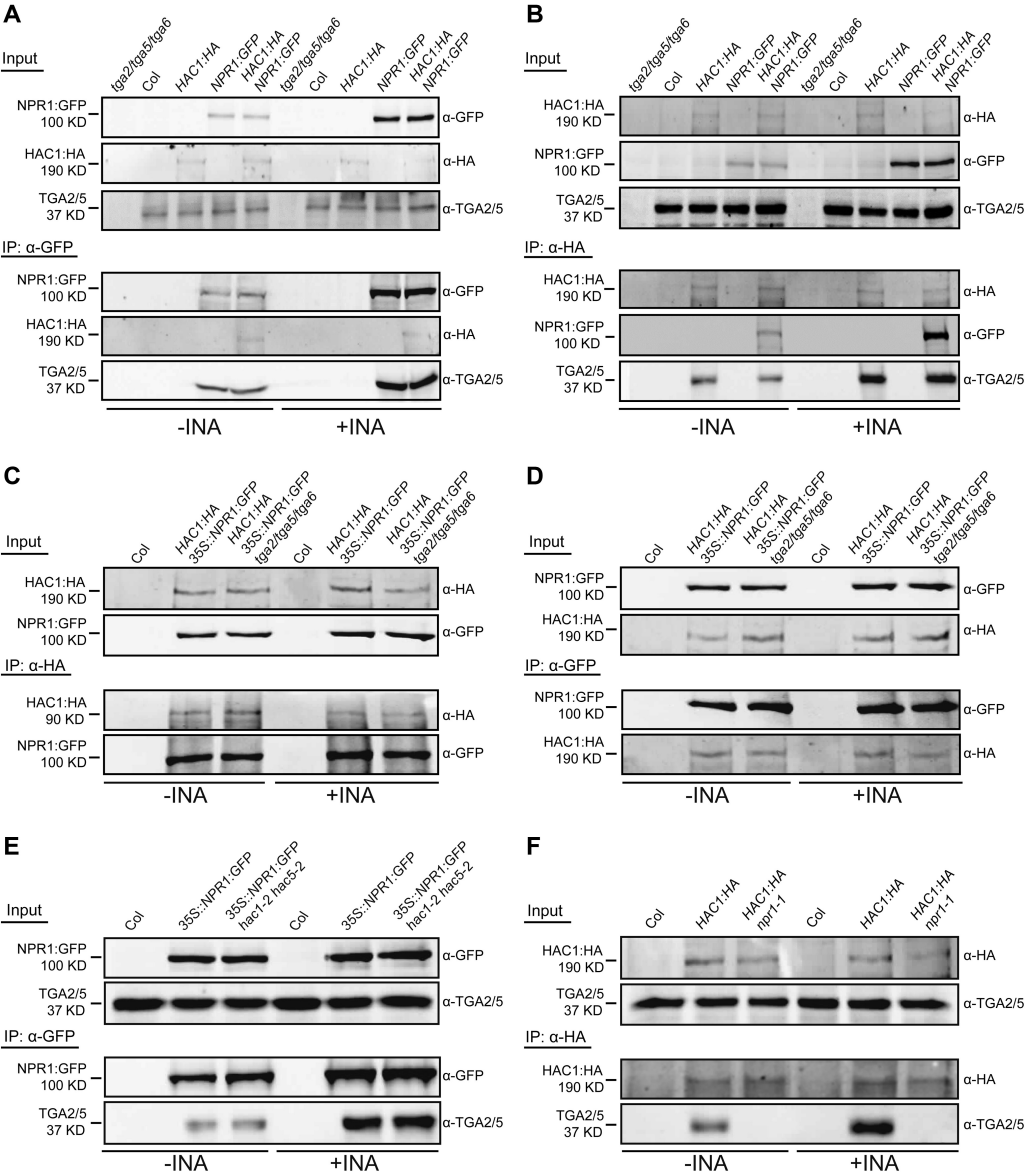
*In vivo* interaction of HAC1 with NPR1 and TGA2/5. (**A**–**F**) co-IP analyses showing the interaction of HAC1 with NPR1 and TGA2/5 (A and B), TGA2/5/6-independent HAC1–NPR1 interaction (C and D), HAC1/5-independent NPR1–TGA2/5 interaction (E) and NPR1-dependent HAC1–TGA2/5 interaction (F). Proteins prepared from DW- or INA-treated plants were IPed and immunoblotted with indicated antibodies. Col and *tga2/5/6* were used as negative controls for co-IP assays. All plants were grown on MS medium for 4 w under short-day condition (8-h light/16-h dark photoperiod) and treated with DW or INA for 12 h before harvest.

To study whether HAC1, NPR1 and TGAs interact with each other, and, if they do, how the SA signal affects their interactions, we examined the subcellular localization of each protein and their interactions before and after INA treatment using stable transgenic *Arabidopsis* plants. HAC1 and TGA2/5 were always localized within nucleus, whereas the abundance and localization of NPR1 were affected by INA (Supplementary Figure S7) as previously reported (12,13,16). HAC1 and TGA2/5 were detected in the NPR1:GFP immunoprecipitate, and reciprocally NPR1 and TGA2/5 were also detected in the HAC1:HA immunoprecipitate (Figure 3A and B; Supplementary Figure S8), revealing the existence of a complex containing HAC1, NPR1 and TGA2/5. TGA2/5, but not HAC1 enrichment within NPR1:GFP immunoprecipitate, was increased by INA (Figure 3A and Supplementary Figure S8A), suggesting that HAC1 might be limiting in complex formation. In contrast, both NPR1 and TGA2/5 enrichments within the HAC1:HA immunoprecipitate were increased by INA (Figure 3B and Supplementary Figure S8B), and similar increases were also observed after pathogen attack (Supplementary Figure S9), implying the possibility of one HAC1 molecule engaging multiple NPR1 and TGA2/5 molecules as SA-bound nuclear NPR1 level increases in response to SA or pathogen signal. This model might be a reminiscent of the interaction of p300 and MEF2 on DNA in which the highly conserved TAZ domain of p300 binds to three MEF2:DNA complexes (55). We could observe interactions between the two TAZ domains of HAC1 and the C-terminal region of NPR1 in yeast (Supplementary Figure S10), suggesting that, similar to p300, the HAC1 TAZ domains might be important for the assembly of the HAC–NPR1–TGA complex.

We then investigated binding dependencies among the components of the HAC–NPR1–TGA complex during the assembly process through a series of co-immunoprecipitation (co-IP) assays. The HAC1–NPR1 interaction was not affected by TGA2/5/6 deficiency (Figure 3C and D), nor was the NPR1–TGA2/5 interaction affected by HAC1/5 deficiency (Figure 3E). Remarkably, HAC1–TGA2/5 interaction was evidently disrupted by the lack of NPR1 (Figure 3F), indicating that HAC1–TGA2/5 interaction is likely indirect and mediated by NPR1.

**Figure 4.**
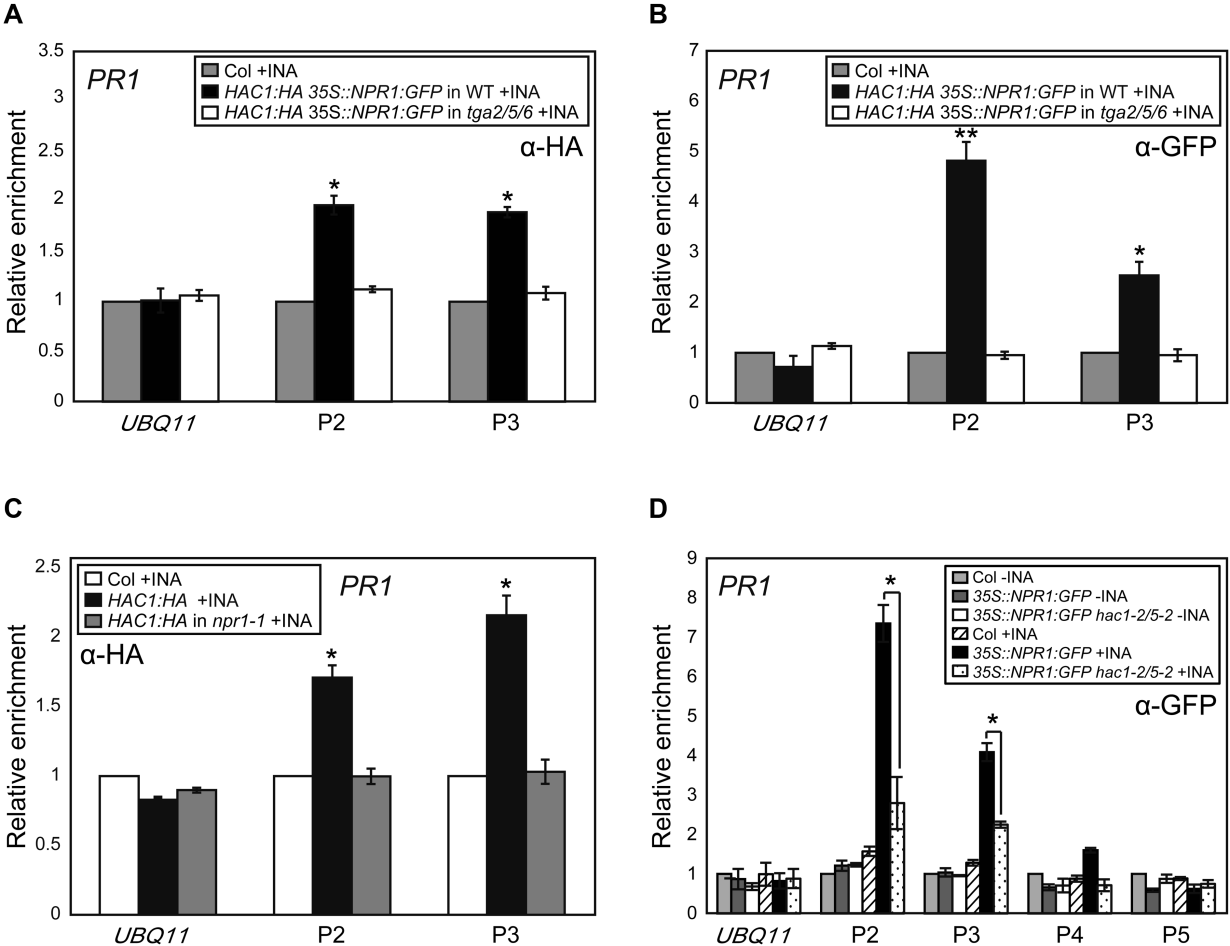
Roles of TGA2/5/6, NPR1 and HAC1/5 in HAC1 and NPR1 targeting to *PR1* chromatin. (**A** and **B**) ChIP assays showing TGA2/5/6-dependent INA-induced association of HAC1:HA (A) and NPR1:GFP (B) with *PR1* chromatin. (**C**) NPR1-dependent targeting of HAC1 to *PR1*. (**D**) Reduced NPR1 targeting to *PR1* by *hac1/5* mutations. ChIP-qPCR was performed with indicated antibody and the level of untagged (A–C) or untagged and untreated Col (**D**) was set to 1 after normalization by corresponding input. Shown are means ±SE of three independent ChIP experiments performed in triplicates. Asterisks indicate statistically significant differences compared to Col+INA (A–C) or *35S::NPR1:GFP*+INA (D) (*P < 0.05 and **P < 0.01 in a Student’s *t*-test). All plants were grown on MS medium for 4 w under short-day condition (8-h light/16-h dark photoperiod) and treated with DW or INA for 12 h before harvest.

ChIP assays were then used to study the binding hierarchy of HAC, NPR1 and TGA to *PR1* chromatin. INA-induced targeting of NPR1 and HAC1 to *PR1* chromatin was completely abolished in *tga2/5/6* triple mutants (Figure 4A and B), and notably, INA-induced HAC1 targeting to *PR1* was undetectable in *npr1* mutants (Figure 4C). These results point to that HAC1 and NPR1 are recruited to *PR1* chromatin via the interaction between NPR1 and the DNA-binding protein TGA as expected from the co-IP results (Figure 3A**–**F). Strikingly, in contrast to the HAC1/5-independent NPR1–TGA2/5 interaction (Figure 3E), NPR1 but not TGA2 targeting was reduced largely in *PR1* and to a lesser extent in *PR2* chromatin in the absence of HAC1/5 (Figure 4D and Supplementary Figure S11A–C). Therefore, although HAC1/5 may not be required for the interaction between NPR1 and free TGAs, they are likely required for efficient NPR1 binding to TGAs in the chromatin context. One possibility is that HAC recruited via NPR1 to *PR* chromatin might modify local chromatin landscape by acetylating histones, which in turn might allow more stable association of the HAC–NPR1–TGA complex with chromatin. Alternatively, HAC might act as an adaptor forming multivalent interactions with transcription factors and thus stabilizing NPR1 association with *PR* chromatin. It is also possible that SA-binding to NPR1 might induce a conformational change to the HAC–NPR1 complex or to the ternary HAC–NPR1–TGA complex rendering more efficient interaction with DNA-bound TGAs or *PR* promoters. In sum, one role of HAC might be to facilitate or/and stabilize the establishment of the functional HAC–NPR1–TGA complex on *PR* chromatin.

### HACs are essential components of the SA-induced NPR1- and TGA-containing high molecular-weight complex

**Figure 5.**
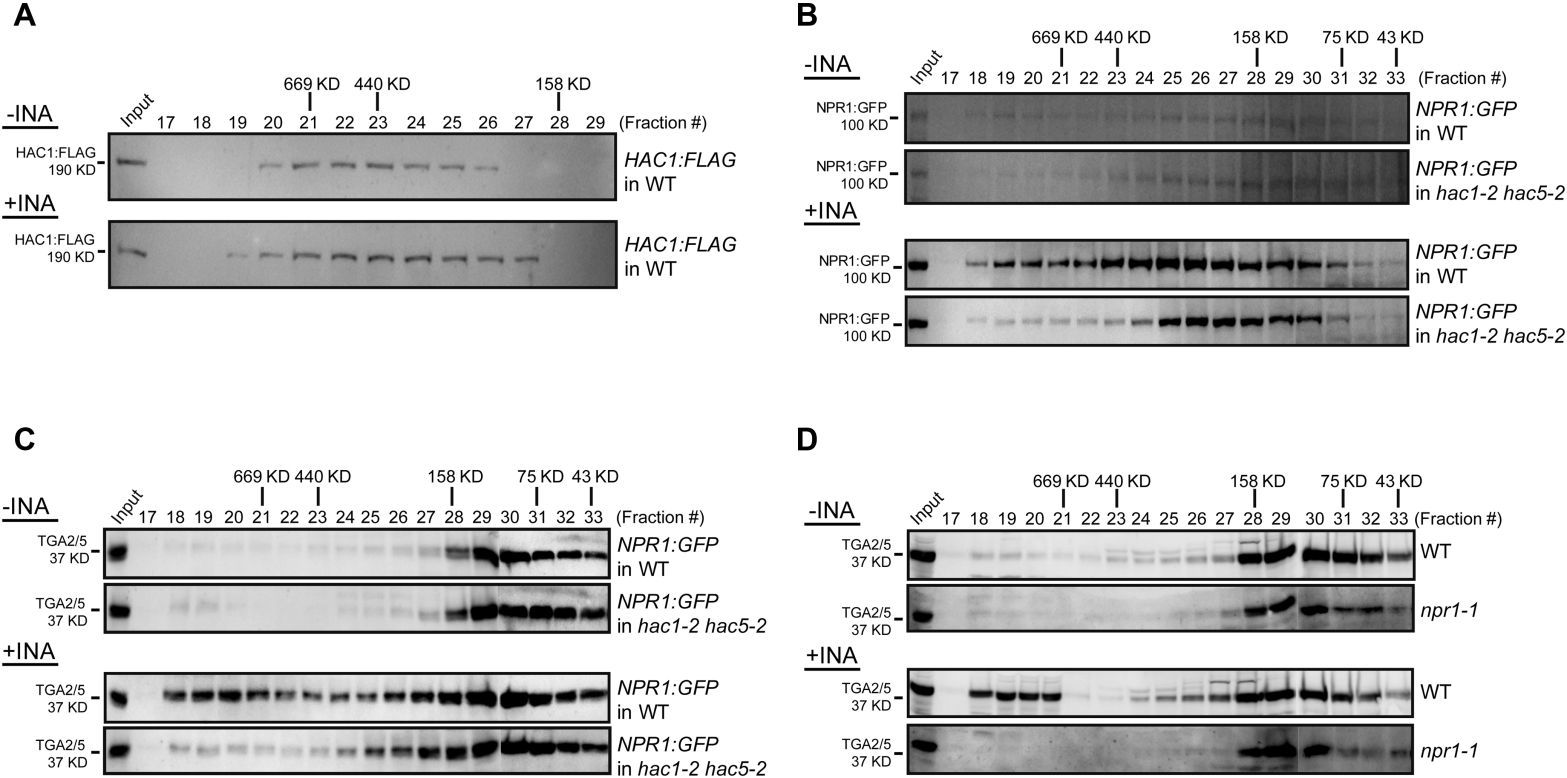
Fractionation of the HAC–NPR1–TGA complex. (**A**–**D**) Immunoblot analysis of FPLC fractions. Proteins from *HAC1:FLAG* (A), *NPR1:GFP* or *NPR1:GFP* in *hac1/5* (B and C) and WT or *npr1* (D) plants were fractionated by FPLC and subjected to immunoblot analyses with indicated antibodies. Molecular-weight standards used (thyroglobulin (669 KD), ferritin (440 KD), aldolase (158 KD), conalbumin (75 KD) and ovalbumin (43 KD)) were co-fractionated with proteins. Plants were grown on MS medium for 4 w under short-day condition (8-h light/16-h dark photoperiod) and treated with DW or INA for 12 h before harvest.

To gain further insight into the HAC–NPR1–TGA complex *in vivo*, we performed gel-filtration chromatography assays. Without INA treatment, HAC1:FLAG, NPR1:GFP and TGA2/5 were predominantly identified in fractions with molecular weights greater than their respective predicted monomeric sizes (Figure 5A–D and Supplementary Figure S12), suggesting their presence within complexes *in vivo* (12,13,54). Noticeably, INA treatments broadened and shifted the elution profiles of HAC1:FLAG toward both larger and smaller mass ranges (Figure 5A). NPR1:GFP expressed by the native promoter of *NPR1* was greatly increased in abundance by INA treatment in all fractions where NPR1:GFP was detected (Figure 5B). When NPR1:GFP was overexpressed, its elution profile was clearly shifted toward larger mass ranges by INA treatment, although its abundance was not greatly increased (Supplementary Figure S12A). INA treatment also substantially affected the elution profile of TGA2/5 to form another peak at much higher mass range (∼fraction #19 in Figure 5C and D; Supplementary Figure S12B), resulting in the co-presence of HAC1:FLAG, NPR1:GFP and TGA2/5 in fractions >669 KD range. Thus, by SA signal HAC1, NPR1 and TGA2/5 may form a >669 KD multiprotein complex.

For deeper understanding of the role of HACs in the assembly of the HAC–NPR1–TGA complex, we then compared the elution profiles of NPR1:GFP and TGA2/5 in WT versus *hac1/5* mutants (Figure 5B and C). Without INA, the elution profiles of NPR1:GFP and TGA2/5 were similar between WT and *hac1/5*. However, after INA treatment, the abundance of NPR1:GFP in higher molecular-weight fractions was evidently decreased by *hac1/5* mutations (Figure 5B and Supplementary Figure S12A). Furthermore, TGA2/5 abundance in fractions >669 KD was also drastically reduced or eliminated in *hac1/5* mutants and instead TGA2/5 were detected in smaller-weight fractions (Figure 5C and Supplementary Figure S12B). Thus, HACs are essential components of the INA-induced high molecular-weight complex containing NPR1 and TGAs. Similarly, after INA treatment, TGA2/5 were not detected in the >669 KD fractions in *npr1* mutants (Figure 5D), consistent with the co-IP results showing NPR1-dependent HAC1–TGA2/5 interaction (Figure 3F).

### Several hundred genes are commonly regulated by NPR1 and HAC1/5

**Figure 6.**
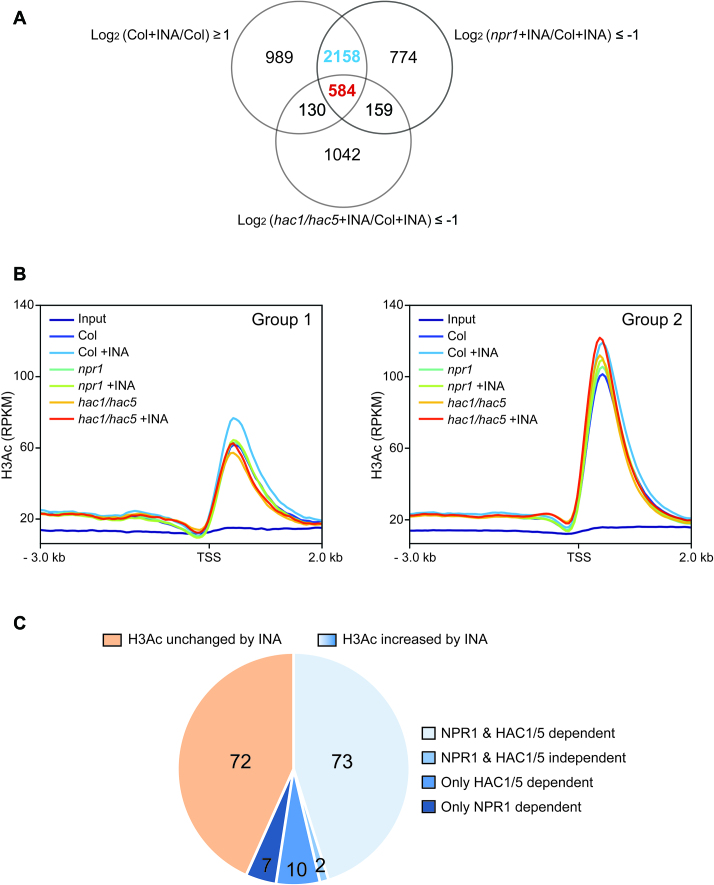
Role of NPR1 and HAC1/5 in INA-induced transcriptome and genome-wide H3Ac dynamics. (**A**) Venn diagram illustrating number of genes induced by INA (FDR ≤ 0.2) as identified through RNA seq. The number of genes co-regulated by NPR1 and HAC1/5 (Group 1) or regulated by NPR1 only (Group 2) is indicated by red or blue, respectively. (**B**) Distribution of H3Ac at the Group 1- and Group 2-gene loci. H3Ac level is presented as RPKM of reads from ChIP-seq data. Region from the 3 kb upstream to the 2 kb downstream of transcription start site (TSS) was scanned with 50 bp interval. (**C**) Pie-chart showing the proportion of further selected Group 1-gene loci (see the text) with or without increased H3Ac after INA treatment. Region from the 2 kb upstream of TSS to the 1 kb downstream of transcription termination site was considered for H3Ac level. Plants were grown on soil for 4 w under short-day condition (8-h light/16-h dark photoperiod) and treated with DW or INA for 12 h before harvest.

To assess the role of the collaboration between NPR1 and HACs in SA-induced transcriptional reprogramming at genome-wide level, we performed RNA-seq analyses of transcriptomes of WT, *npr1* and *hac1/5* either treated with INA or not (Figure 6A). About 71% and 18% of the genes significantly upregulated by INA in WT were not upregulated in *npr1* and *hac1/5*, respectively. Among the NPR1-dependent genes (2742), 21% (584) also showed HAC1/5-dependency (Group 1; Supplementary Datasets S1 and S2), whereas the remaining 79% (2158) did not (Group 2; Supplementary Datasets S1 and S2). The RNA-seq results were confirmed by RT-qPCR analyses of dozens of randomly selected genes from each group (Supplementary Figures S13 and S14). Thus, a small but considerable fraction (15%) of the INA-induced transcriptome in WT is dependent on both NPR1 and HAC1/5 (Group 1), whereas a larger fraction (56%) requires only NPR1 (Group 2).

### HACs also regulate SA biosynthesis or accumulation-related genes in an NPR1-independent manner

Despite relatively small portion of the Group 1 genes, the susceptibilities of *hac1/5* and *npr1* against *Pst* DC3000 were comparable (Figure 2F). This suggests that the genes co-regulated by HAC1/5 and NPR1 might be crucial in plant immunity. Alternatively, HACs might affect plant immunity in an NPR1-independent as well as NPR1-dependent manners. Lately, it was reported that the ethylene-signaling pathway is hyper-activated in *hac1/5* (33). As ethylene (Et) and jasmonic acid (JA) antagonistically crosstalk with SA in general (56–59), the activation of Et/JA-signaling could suppress the SA-dependent defense pathway (60). Thus, first we compared the expressions of *ERF1* and *ERF2* (56,61), genes encoding ethylene-response factors and *CHIB* and *VSP2* (62), the JA- and Et-signaling pathway markers, respectively, in WT, *npr1* and *hac1/5* (Supplementary Figure S15A). Although, the expression levels of *ERF1* and *ERF2* in *hac1/5* were higher than in WT and *npr1* after and before *Pst* DC3000 infection, respectively, pathogen-induced expressions of *CHIB* and *VSP2* were significantly reduced in *hac1/5* but not in *npr1*, indicating that the susceptibility of *hac1/5* was not likely caused by the activated Et/JA pathways.

We then examined the effect of *hac1/5* mutations on the pathogen-induced expression of several SA biosynthesis or accumulation-related genes, namely *ICS1* (63), *EDS5* (64), *PAD4* (65) and *GDG1* (66,67) as the induction of *ICS1* and *EDS5* by pathogen infection was known to be NPR1-independent (68–70). Induction of *ICS1, EDS5* and *PAD4* by *Pst* DC3000 were significantly reduced by *hac1/5*, but not by *npr1* mutations (Supplementary Figure S15B). Further, pathogen-induced targeting of HAC1:HA but not NPR1:GFP was observed in the examined regions of *ICS1* and *EDS5* promoters (Supplementary Figure S16). These results are consistent with the previously reported NPR1-independent pathogen-induced expression of these genes and indicate that HACs also promote SA-dependent immunity by NPR1-independently regulating SA biosynthesis or accumulation-related genes, explaining part of the severe pathogen-susceptible phenotype of *hac1/5*.

### HACs are epigenetic partners of NPR1 and the HAC–NPR1–TGA complex constitutes part of the genome-wide SA-induced transcriptional reprogramming system

Next, by ChIP seq we studied how H3Ac levels are affected by INA, *npr1* and *hac1/5* mutations at the Group 1- and Group 2-gene loci (Figure 6B). Reproducibility of the ChIP-seq data was confirmed by ChIP-qPCR analyses of 11 selected loci (Supplementary Figure S17). At the Group 1-gene loci, H3Ac levels at the downstream of the transcription start sites were substantially increased by INA treatment in WT. However, this INA-induced increase was not observed both in *npr1* and *hac1/5* mutants. The Group 2-gene loci showed ∼1.5-fold higher basal H3Ac levels and clear but less substantial INA-induced H3Ac increase compared to the Group 1-gene loci in WT. In *hac1/5* mutants, the INA-induced H3Ac increase was still obvious at the Group 2-gene loci even with higher basal level than WT. However, the INA-induced H3Ac increase at the Group 2-gene loci was almost fully abolished by the *npr1* mutation as at the Group 1-gene loci. Thus, in line with the HAC1/5- as well as NPR1-dependent INA-induced expression pattern, the Group 1 genes show both NPR1- and HAC1/5-dependent INA-induced H3Ac increase on average. Furthermore, consistently with the NPR1-, but not HAC1/5-dependent INA-induced expression pattern, the Group 2 genes show stronger dependency on NPR1 than HAC1/5 in INA-induced H3Ac increase as well.

Then, to examine the role of NPR1 and HAC1/5 in the INA-induced H3Ac increase at individual Group 1-gene loci, first we further selected Group 1 genes that show heavier INA, NPR1 and HAC1/5 dependency by using stricter criteria: log_2_[(Col+INA)/Col] ≥ 2, log_2_[(*npr1*+INA)/(Col+INA)] ≤ -2, log_2_[(*hac1/5*+INA)/(Col+INA)] ≤ -2 and FDR ≤ 0.05 (Figure 6C and Supplementary Datasets S3–S6). At 56% of the further selected loci, H3Ac levels were substantially increased by INA, and 79% of these loci showed compromised H3Ac increases in both *npr1* and *hac1/5* mutants. Considering diverse mechanisms other than histone acetylation for the transcriptional coactivator role of CBP/p300 HATs as mentioned in the Introduction, we believe it is plausible that at least part of the Group 1 genes are expected to show no good correlations between H3Ac and RNA expression, even though they are the direct targets of HACs and NPR1. Taken all together, our ChIP-seq and RNA-seq analyses indicate that the HAC–NPR1–TGA complex constitutes part of the genome-wide transcriptional activator system responsible for the SA-induced transcriptional reprogramming.

## DISCUSSION

**Figure 7.**
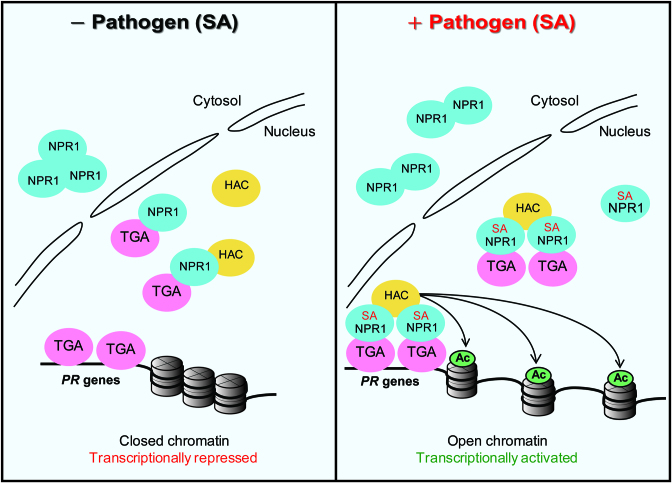
Model for the epigenetic reprogramming of *PR* genes by the HAC–NPR1–TGA complex. Under normal condition (left), NPR1 (blue oval) preferentially presents within the cytoplasm as oligomers while its minor fraction is within the nucleus and interacts with TGA (pink oval) and HAC (yellow oval). Although TGA, which is not in the ternary HAC–NPR1–TGA complex, binds to *PR* promoters and represses *PR* transcription, the HAC–NPR1–TGA complex is not recruited to *PR* chromatin under this condition. Upon pathogen challenge and following SA surge (right), the nuclear fraction of NPR1 is increased due to enhanced stability and translocation of part of the cytoplasmic NPR1 into the nucleus. With increased concentration and SA binding, NPR1 interacts with HAC possibly in a multiple:one fashion. The SA-bound HAC–NPR1–TGA complex is now recruited to *PR* promoters or alternatively the SA-bound HAC–NPR1 complex is recruited by TGA on *PR* promoters, and the ternary complex induces transcriptional activation through histone acetylation (Ac)-dependent chromatin reprogramming. SA might induce a conformation change to the HAC–NPR1–TGA complex and activate it during this process.

Although NPR1 is a well-known master regulator of the SA-dependent immunity and systemic acquired resistance, how it acts as a transcriptional coactivator for over 2000 downstream genes is not fully understood at the molecular level. Our study demonstrates a molecular mechanism for the coactivator role of NPR1 in which NPR1 acts in concert with HACs as epigenetic partners and that the HAC–NPR1–TGA complex is involved in genome-wide transcriptional reprogramming through histone acetylation-based mechanism (Figure 7). Interestingly, the SA-induced transcriptional reprogramming model we propose here is a reminiscent of the steroid hormone-induced epigenetic and transcriptional reprogramming model prevalent in animal systems. It is also possible that HAC in the ternary complex might also acetylate transcriptional regulators including NPR1/TGAs and affect their transcription activities or might act as a scaffold leading to the formation of a large transcription-activator complex required for *PR* expression. Further, our work indicates that epigenetic reprogramming is a central feature of the immune system in plants which, unlike animals, lack specialized immune cells.

Our finding of both HAC-dependent (Group 1) and independent (Group 2) NPR1-regulated genes suggests that NPR1 might act in different modes depending on target chromatin contexts. For example, the degree of chromatin compaction could be a factor in the HAC requirement. Our finding of higher basal H3Ac levels at the Group 2-gene loci compared to the Group 1-gene loci (Figure 6B) supports this hypothesis. The Group 2 genes might be in open chromatin conformation with enriched basal H3Ac and poised to respond to SA-activated NPR1 without assistance from HATs. In this case, the INA-induced increased H3Ac levels observed in WT and *hac1/5* might be consequences rather than causes of the increased transcriptional activities in those plants. Alternatively, HATs other than HACs might act as epigenetic partners of NPR1 for the Group 2 genes. The Group 1 and Group 2 genes do not seem to act in different biological processes as our preliminary gene ontology analysis did not reveal significant differences between them. Thus, it would be of interest in the future to understand the chromatin features of the Group1 and Group 2 genes or the dependency of the Group 2 transcription on chromatin factors other than HACs. Comprehensive evaluations on the genome-wide role of the HAC–NPR1–TGA complex in SA-induced transcriptional reprogramming will be possible through integrative analyses of data from RNA seq, H3Ac ChIP seq and the genome-wide association studies of HACs, NPR1 and TGAs.

## DATA AVAILABILITY

The ChIP-seq and RNA-seq data have been deposited in the Gene Expression Omnibus (GEO) under the SuperSeries accession number GSE101572. All other data are available from the authors upon reasonable request.

## Supplementary Material

Supplementary Data
