# Supplementary material for: Salicylic acid-induced transcriptional reprogramming by the HAC–NPR1–TGA histone acetyltransferase complex in Arabidopsis
Source: Nucleic Acids Res. 2018 Sep 17;46(22):11712–25. doi: 10.1093/nar/gky847 (PMC6294559; doi:10.1093/nar/gky847)
Supplement: Supplementary Data [file gky847_supplemental_files.zip › Jin_Supplementary Data.pdf]

SUPPLEMENTARY DATA

Salicylic acid-induced transcriptional reprogramming by the HAC-NPR1-TGA histone acetyltransferase complex in *Arabidopsis*

Hongshi Jin[1]†, Sun-Mee Choi[1]†, Min-Jeong Kang[1], Se-Hun Yun[1], Dong-Jin Kwon[1], Yoo-Sun Noh[1,2]* & Bosl Noh[3]*

[1]School of Biological Sciences, Seoul National University, Seoul 08826, Korea, [2]Plant Genomics and Breeding Institute, Seoul National University, Seoul 08826, Korea, [3]Research Institute of Basic Sciences, Seoul National University, Seoul 08826, Korea

*To whom correspondence should be addressed. Tel: +82 2 871 6675; Fax: +82 2 871 6673; Email: bnoh2003@gmail.com
Correspondence may also be addressed to: Yoo-Sun Noh. Tel: +82 2 880 6674; Fax: +82 2 871 6673; ysnoh@snu.ac.kr

†These authors contributed equally to this work.

- Supplementary Figures S1 to S17

- Supplementary Tables S1 to S5

A

HAT family	Arabidopsis gene name	Gene ID
CBP	*HAC1*	At1g79000
	HAC2	At1g67220
	HAC4	At1g55970
	HAC5	At3g12980
	HAC12	At1g16710
GNAT	*HAG1*	At3g54610
	HAG2	At5g56740
	HAG3	At5g50320
MYST	*HAM1*	At5g64610
	HAM2	At5g09740
TAFII250	*HAF1*	At1g32750
	HAF2	At3g19040

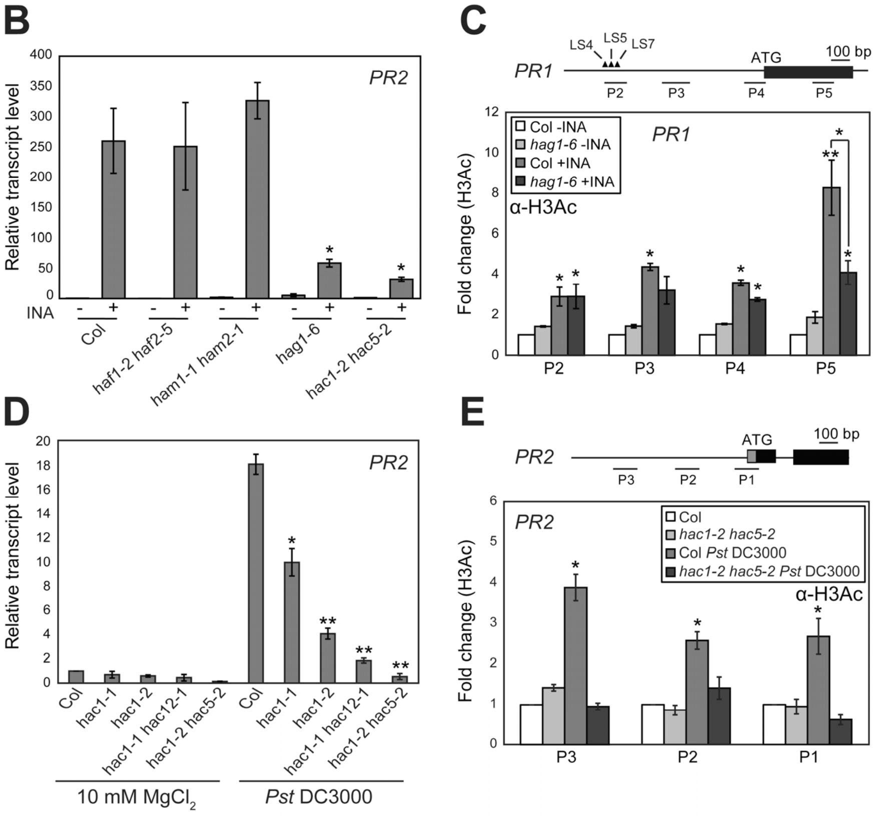

Supplementary Figure S1. HAC1/5 regulate pathogen-induced *PR2* transcription and histone acetylation. (**A**) Canonical HAT-family genes in *Arabidopsis thaliana.* (**B**) *PR2* transcript levels in Col and various HAT mutants treated with INA or not. (**C**) H3Ac levels at *PR1* in Col and *hag1-6* either treated with INA or not. (**D** and **E**) *PR2* transcript levels (**D**) and H3Ac levels at *PR2* (**E**) in Col and *hac* mutants after *Pst* DC3000 infection. Schematics of *PR1* and *PR2* show the regions tested for ChIP-qPCR (**C** and **E**). See Figure 1A for the explanation of the schematics. Means ± SE of three biological experiments performed in triplicates are shown after normalization to *UBQ10* (**B** and **D**) or to input and untagged Col levels (**C** and **E**). Asterisks indicate statistically significant differences compared to Col+INA (**B** and **C**), Col-INA (**C**), infected Col (**D**), or uninfected Col (**E**) (**P* < 0.05 and ***P* <0.01 in a Student's *t*-test). Plants were grown on soil for 4 w under day-neutral condition (12 hr light/12 hr dark photoperiod) and treated with DW or INA for 24 hr before harvest (**B** and **C**), or infected with pathogen for 48 hr before harvest (**D** and **E**).

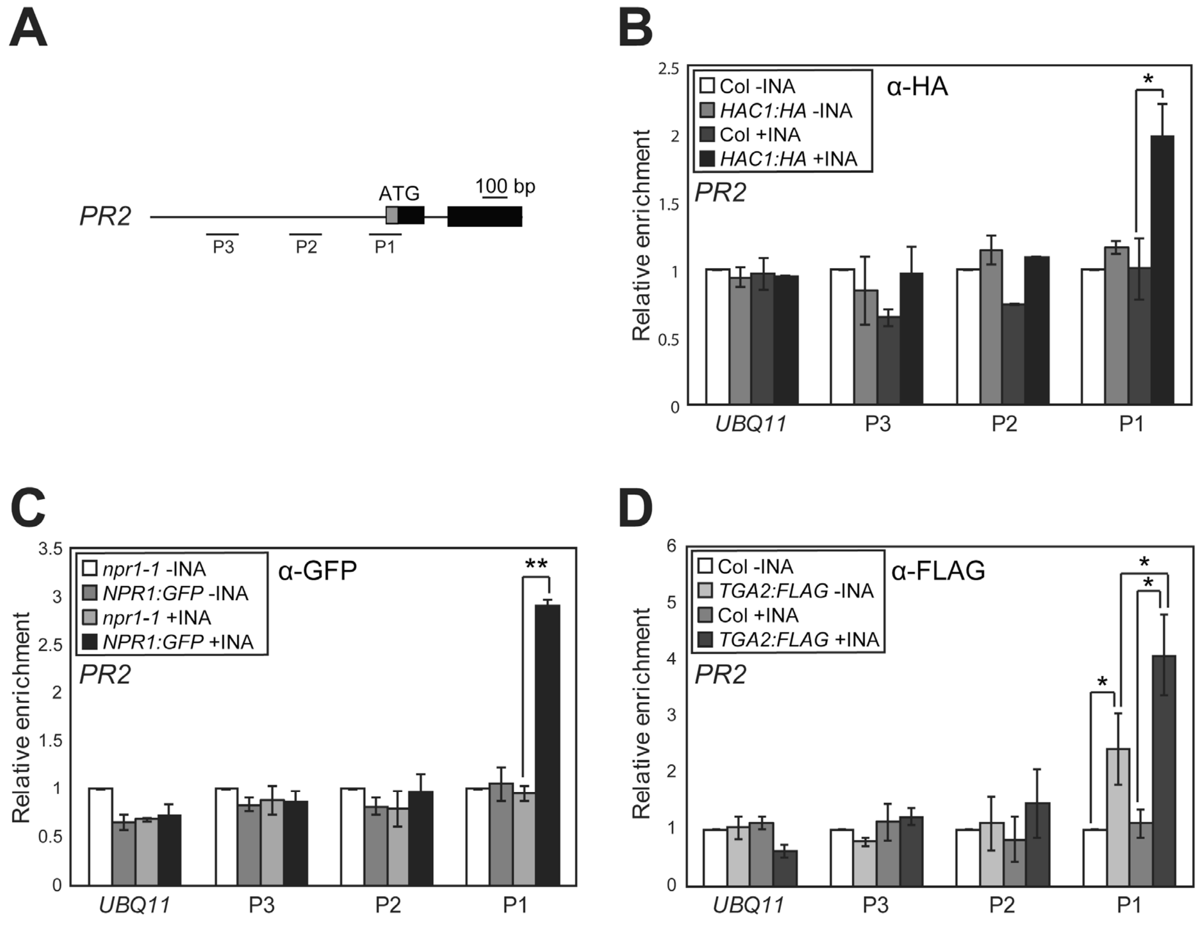

Supplementary Figure S2. Direct targeting of HAC1, NPR1, and TGA2 to *PR*2 chromatin. (**A**) Schematics of *PR2* showing regions tested for ChIP-qPCR. See Figure 1A for the explanation of the schematics. (**B**-**D**) Enrichment of HAC1:HA (**B**), NPR1:GFP (**C**), and TGA2:FLAG (**D**) within *PR2* chromatin. Asterisks indicate statistically significant differences (*P < 0.05 and **P < 0.001 in a Student's *t*-test). Shown are means ± SE of three independent ChIP experiments performed in triplicates. Untreated Col levels were set to 1 after normalization by input. All plants were grown on MS medium for 4 w under short-day condition (8 hr light/16 hr dark photoperiod) and treated with DW or INA for 12 h before harvest.

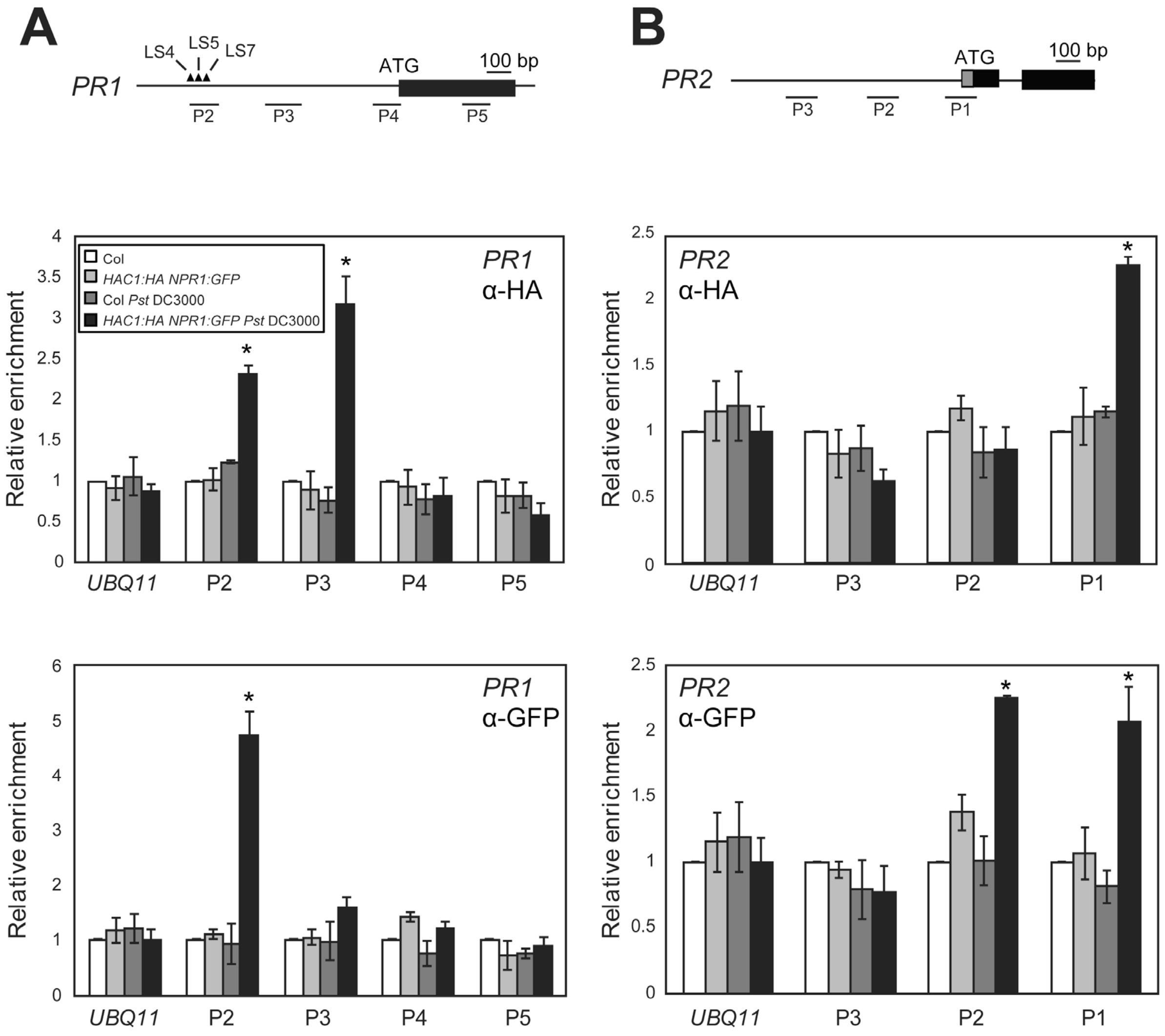

Supplementary Figure S3. HAC1 and NPR1 are targeted to the *PR1* and *PR*2 loci in a pathogen-dependent manner. (**A** and **B**) ChIP assays showing pathogen-dependent targeting of HAC1:HA and NPR1:GFP to *PR1* chromatin (**A**) and *PR2* chromatin (**B**). See Figure 1A for the explanation of the schematics. Anti-HA or anti-GFP antibody was used to detect HAC1:HA and NPR1:GFP enrichment, respectively. Asterisks indicate statistically significant differences ($P <$ 0.05 in a Student's *t*-test) between infected Col and *HAC1:HA NPR1:GFP*. Shown are means ± SE of three independent ChIP experiments performed in triplicates. Uninfected Col levels were set to 1 after normalization by input. Plants were grown on soil for 4 w under day-neutral condition (12 hr light/12 hr dark photoperiod), and leaves were infected with pathogen for 24 hr before harvest.

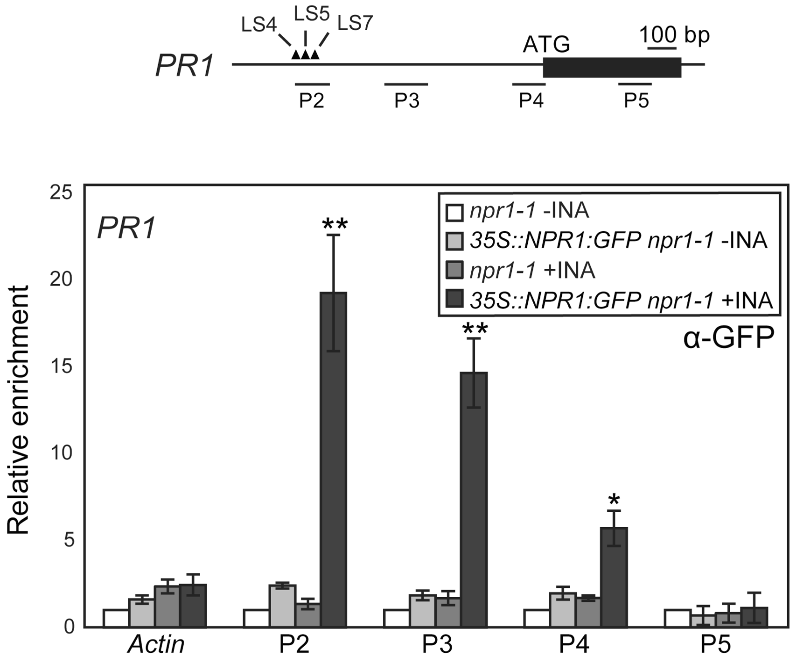

Supplementary Figure S4. NPR1 enrichment within *PR1* chromatin in *35S::NPR1:GFP npr1-1* plants either treated with INA or not. Asterisks indicate statistically significant differences between *35S::NPR1:GFP npr1-1*-INA and *35S::NPR1:GFP npr1-1*+INA (**P* < 0.05 and ***P* < 0.01 in a Student's *t*-test). Shown are means ± SE of three independent ChIP experiments performed in triplicates. Untreated *npr1-1* levels were set to 1 after normalization by input. Plants were grown on MS medium for 4 w under short-day condition (8 hr light/16 hr dark photoperiod) and treated with DW or INA for 12 h before harvest.

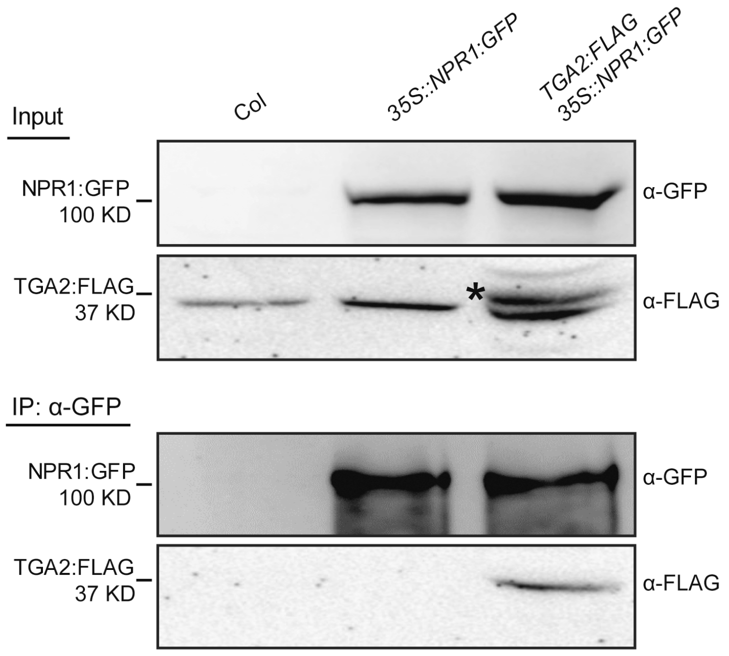

Supplementary Figure S5. TGA2:FLAG forms a complex with NPR1:GFP *in vivo*. Proteins prepared from Col, *35S::NPR1:GFP*, and *TGA2:FLAG 35S::NPR1:GFP* plants were immunoprecipitated with anti-GFP antibody and immunoblotted with anti-GFP or anti-FLAG antibody. An asterisk indicates TGA2:FLAG protein. Plants were grown on MS medium for 4 w under short-day condition (8 hr light/16 hr dark photoperiod), and leaves were harvested for co-IP analysis after 12 hr of INA treatment.

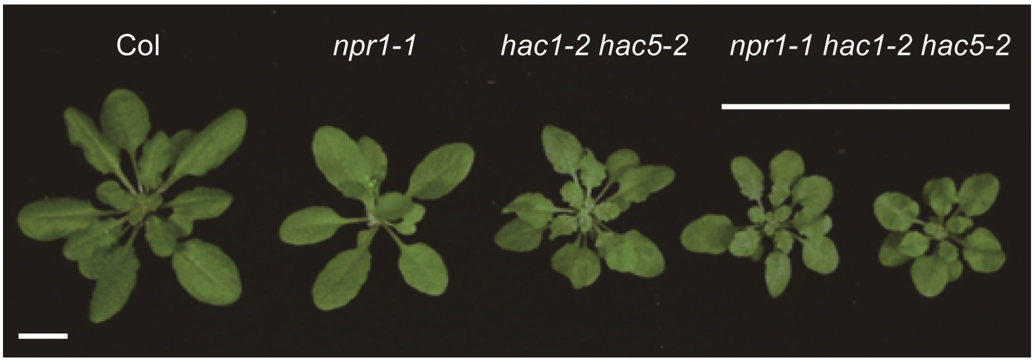

Supplementary Figure S6. Adult phenotypes of Col, *npr1-1*, *hac1-2 hac5-2*, and *npr1-1 hac1-2 hac5-2* mutant plants. Representative plants of each genotype grown for 4 w under day-neutral condition (12 hr light/12 hr dark photoperiod) are shown. Scale bar: 1 cm.

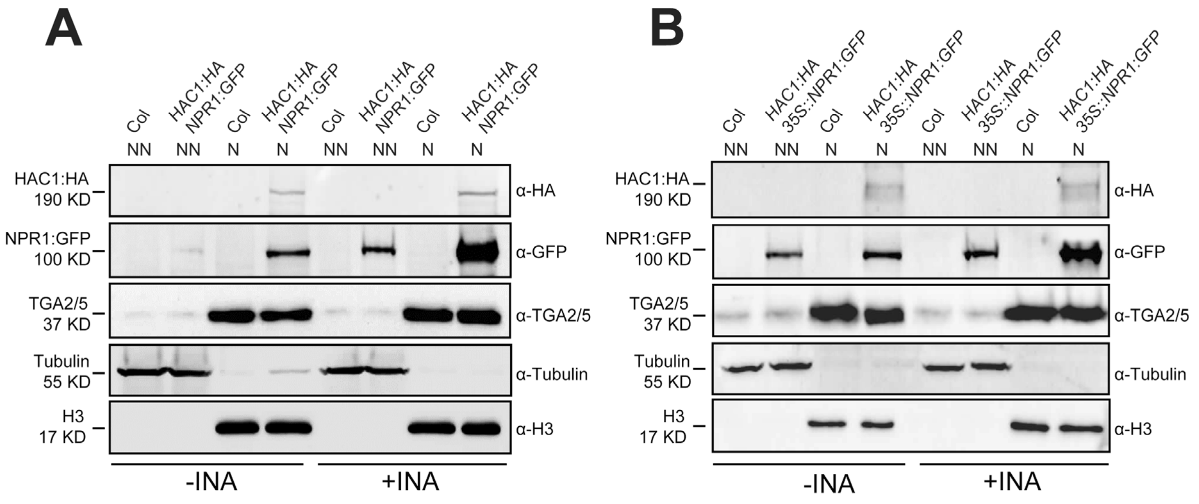

	NN -INA	N -INA	NN +INA	N +INA
NPR1:GFP signal (NN/N ratio)	5.7	1	1.7	1
Nuclear fraction of NPR1:GFP	14.9%		37%	

	NN -INA	N -INA	NN +INA	N +INA
NPR1:GFP signal (NN/N ratio)	14.6	1	6.4	1
Nuclear fraction of NPR1:GFP	6.4%		13.5%	

Supplementary Figure S7. Subcellular localization of HAC1, NPR1, and TGA2/5. (**A** and **B**) Immunoblot analysis of HAC1:HA, NPR1:GFP, and TGA2/5 proteins within nuclear (N) and non-nuclear (NN) fractions using *HAC1:HA NPR1:GFP* (**A**) or *HAC1:HA 35S::NPR1:GFP* (**B**) double transgenic plants. Histone H3 and tubulin were used as nuclear and non-nuclear protein controls, respectively. Table in each panel shows the relative ratios of NPR1:GFP signals on the corresponding immunoblots as quantified by using Image J. NPR1:GFP NN signals were multiplied by 20 as 20 times less NN proteins compared to N proteins were loaded on the gel before immunoblot. NPR1:GFP NN and N signals were normalized by corresponding tubulin and H3 signals, respectively, and the normalized NPR1:GFP N signals were set to 1.

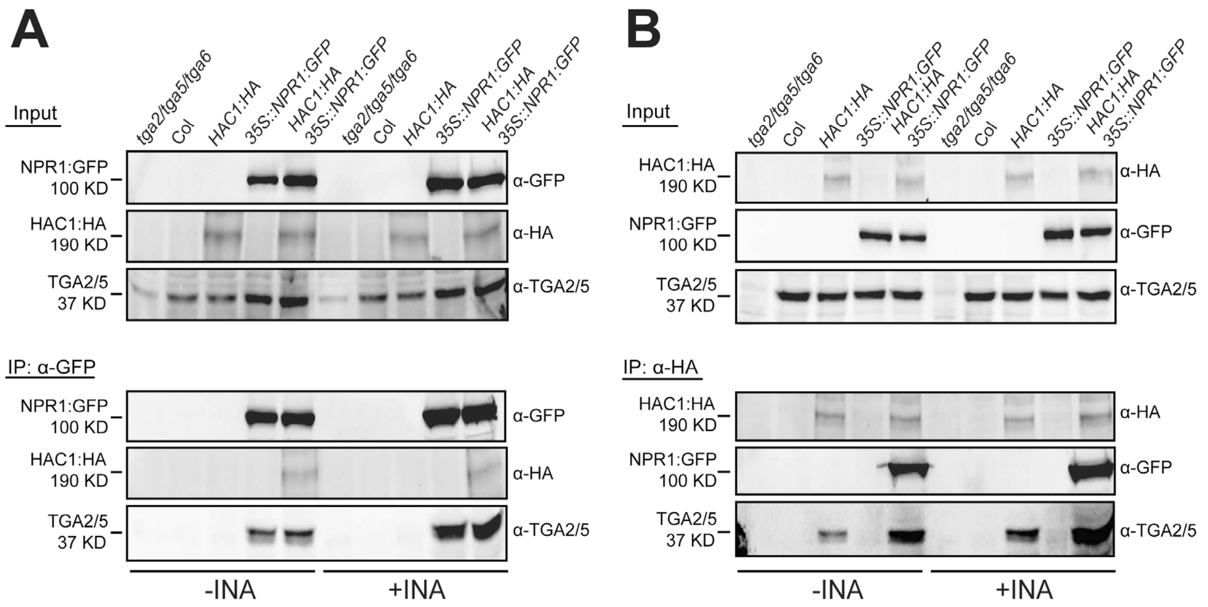

Supplementary Figure S8. *In vivo* interactions among HAC1, NPR1, and TGA2/5. (**A** and **B**) Proteins prepared from *tga2/5/6*, Col, *HAC1:HA*, *35S::NPR1:GFP*, and *HAC1:HA 35S::NPR1:GFP* plants were immunoprecipitated with anti-GFP (**A**) or anti-HA (**B**) antibody and immunoblotted with anti-GFP, anti-HA, or anti-TGA2/5 antibody. Plant samples were prepared as described in Figure 3.

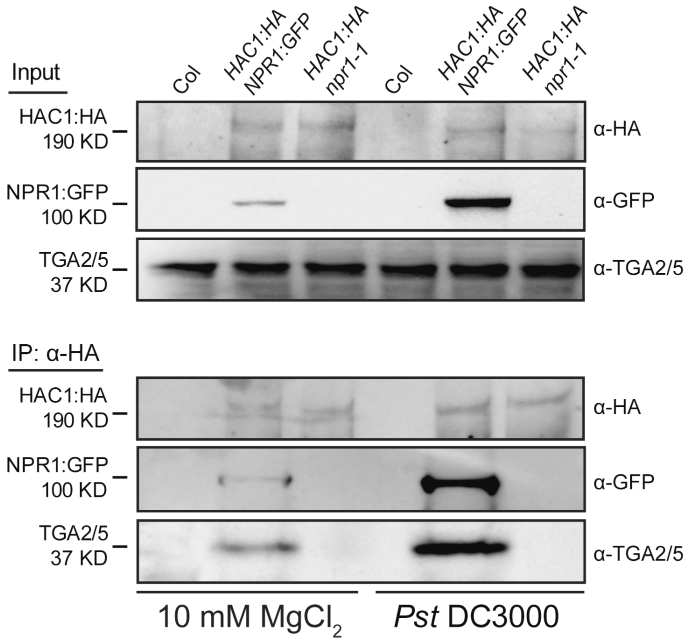

Supplementary Figure S9. *In vivo* interactions among HAC1, NPR1, and TGA2/5 in Col, *HAC1:HA NPR1:GFP*, and *HAC1:HA npr1-1* plants without or with pathogen infection. Proteins were immunoprecipitated with anti-HA antibody and immunoblotted with anti-HA, anti-GFP, or anti-TGA2/5 antibody. Plants were grown on soil for 4 w under day-neutral condition (12 hr light/12 hr dark photoperiod) and harvested 24 hr after injection with 10 mM $MgCl_2$ or pathogen.

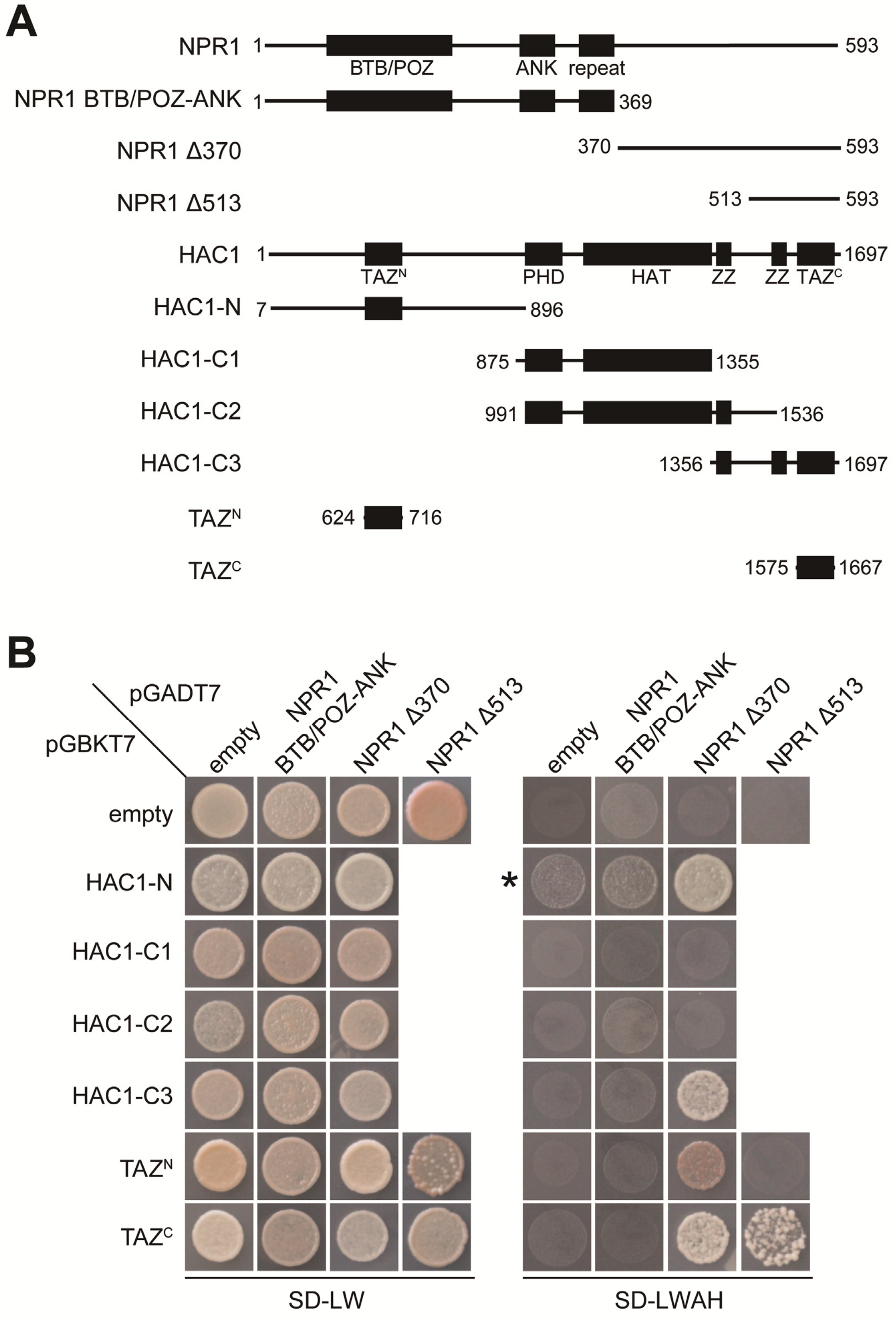
A
NPR1
1
593
BTB/POZ
ANK repeat
NPR1 BTB/POZ-ANK
1
369
NPR1 Δ370
370
593
NPR1 Δ513
513
593
HAC1
1
1697
TAZ^N
PHD
HAT
ZZ
ZZ
TAZ^C
HAC1-N
7
896
HAC1-C1
875
1355
HAC1-C2
991
1536
HAC1-C3
1356
1697
TAZ^N
624
716
TAZ^C
1575
1667
B
pGADT7
pGBKT7
empty
NPR1 BTB/POZ-ANK
NPR1 Δ370
NPR1 Δ513
empty
HAC1-N
HAC1-C1
HAC1-C2
HAC1-C3
TAZ^N
TAZ^C
*
SD-LW
SD-LWAH

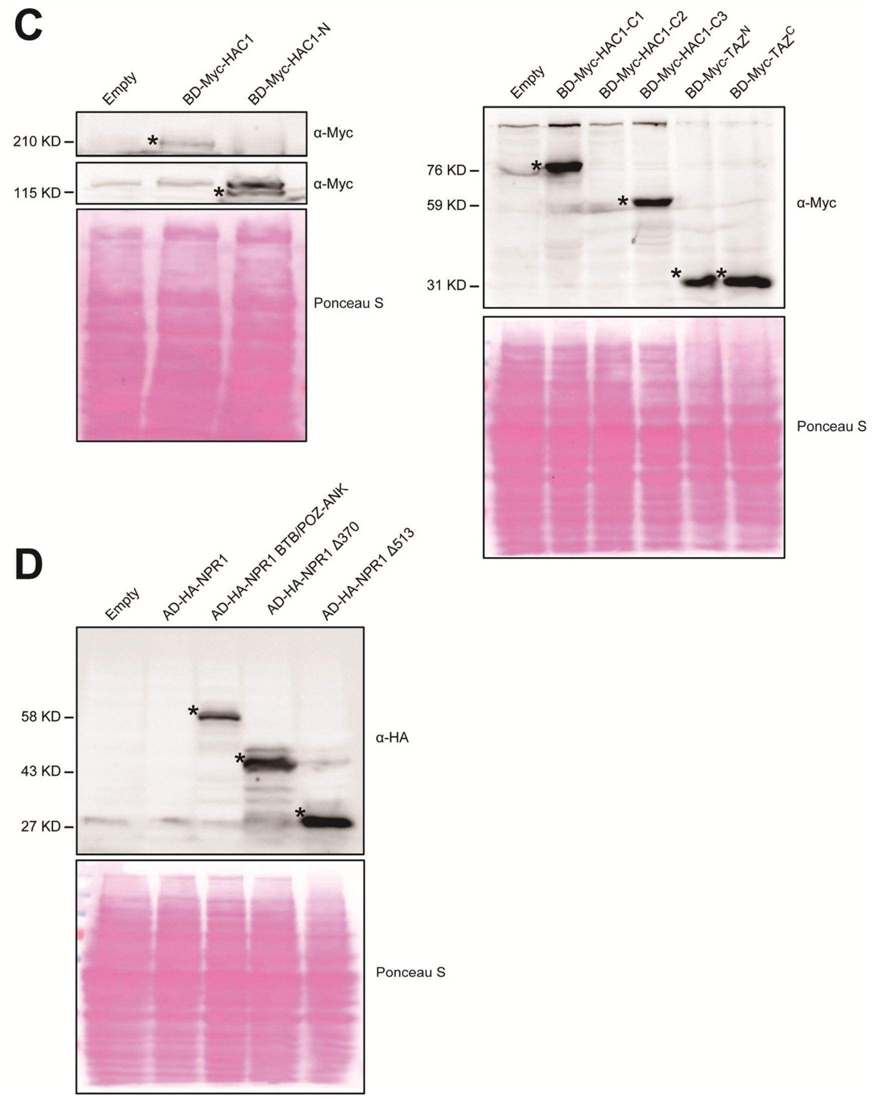

Supplementary Figure S10. Interaction between HAC1 and NPR1 in yeast. (**A**) Schematics of NPR1 and HAC1 deletions used for Yeast-Two-Hybrid assays. HAC1 deletions were fused to the GAL4 DNA-binding domain and the NPR1 deletions were fused to the GAL4 activation domain. (**B**) Yeast transformants were grown on Leu^- Trp^- dropout media (SD-LW) or Leu^- Trp^- $adenine^-$ His^- dropout media (SD-LWAH) containing 1 mM 3-aminotriazole (3-AT) except for the case of HAC1-N as a bait (*) for which 3 mM 3-AT was used. (**C** and **D**) Expression of the bait and prey proteins in yeast. AH109 yeast cells containing the bait and prey were cultured in –LW media to OD_{600} = 0.8~1.0, then total protein was extracted by bead-beating method. Expressions of the BD-Myc-HAC1 and BD-Myc-HAC1-N were detected using yeast cells co-expressing AD-HA-NPR1 and AD-HA-NPR1 BTB/POZ-ANK, respectively. Expressions of the BD-Myc-HAC1-C1, BD-Myc-HAC1-C2/-C3, and BD-Myc-TAZ^N/-TAZ^C were detected using yeast cells co-expressing AD-HA-NPR1 BTB/POZ-ANK, AD-HA-NPR1 Δ370, AD-HA-NPR1 Δ513, respectively. Expressions of the AD-HA-NPR1, AD-HA-NPR1 BTB/POZ-ANK, AD-HA-NPR1 Δ370, and AD-HA-NPR1 Δ513 were detected using yeast cells co-expressing BD-Myc-HAC1, BD-Myc-HAC1-C1, BD-Myc-HAC1-C2, and BD-Myc-TAZ^N, respectively.

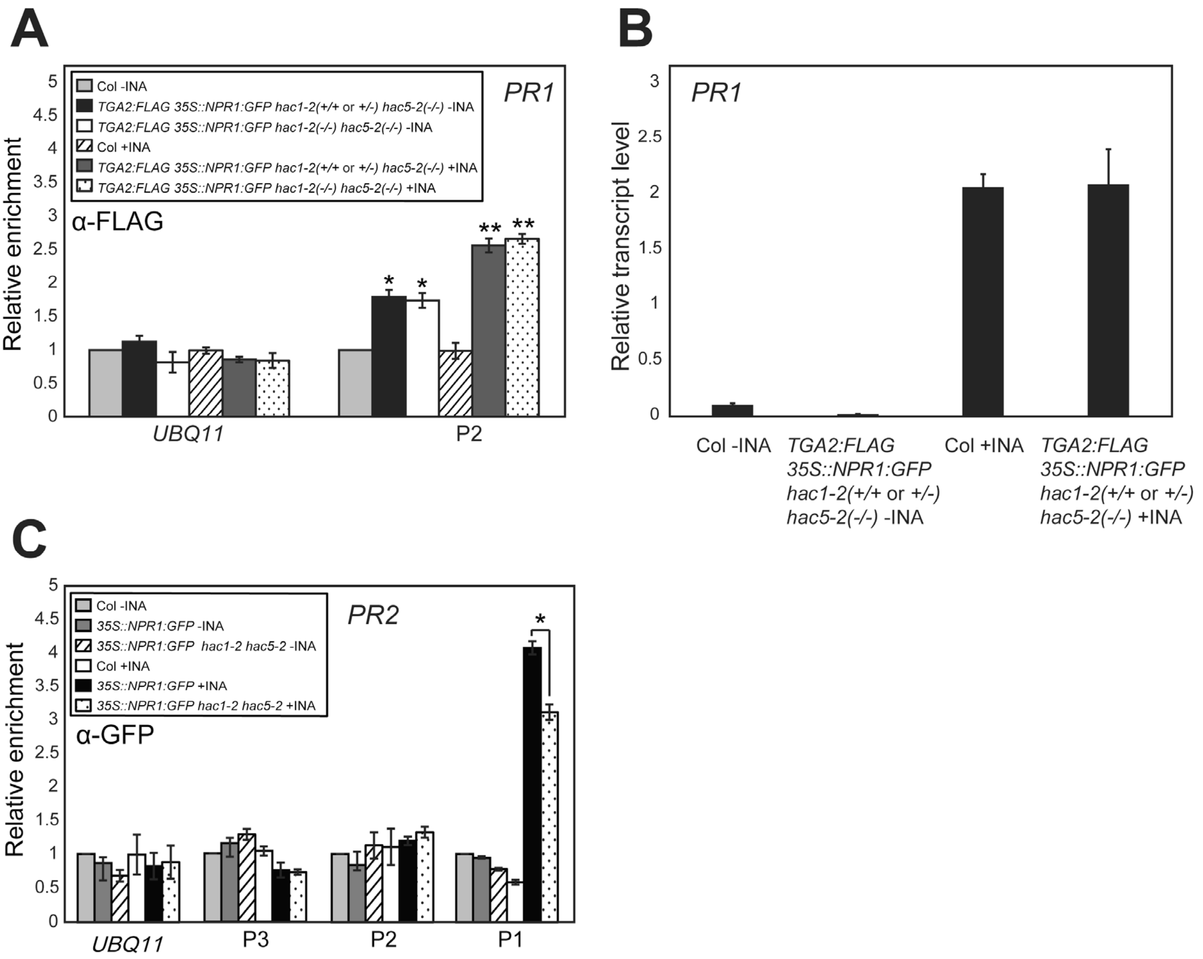

Supplementary Figure S11. Role of HAC1/5 in TGA2 and NPR1 targeting to *PR1* or *PR2* chromatin. (**A**) ChIP assay showing HAC1/5-independent TGA2:FLAG targeting to *PR1* chromatin. (**B**) RT-qPCR analysis of transcript levels of *PR1* in Col and *TGA2:FLAG 35S::NPR1:GFP hac1-2*(+/+ or +/-) *hac5-2*(-/-) plants either treated with INA or not. (**C**) Reduced targeting of NPR1:GFP to *PR2* chromatin by *hac1/5* mutations. Asterisks indicate statistically significant differences compared to Col-INA (**A**) or *35S::NPR1:GFP*+INA (**C**) (*$P < 0.05$ and **$P < 0.01$ in a Student's *t*-test). Shown are means ± SE of three independent experiments performed in triplicates. INA-untreated Col levels were set to 1 after normalization by input (**A** and **C**). Transcript levels were presented after normalization to *UBQ10* (**B**). Plants were grown on MS medium for 4 w under short-day condition (8 hr light/16 hr dark photoperiod) and treated with DW or INA for 12 hr before harvest.

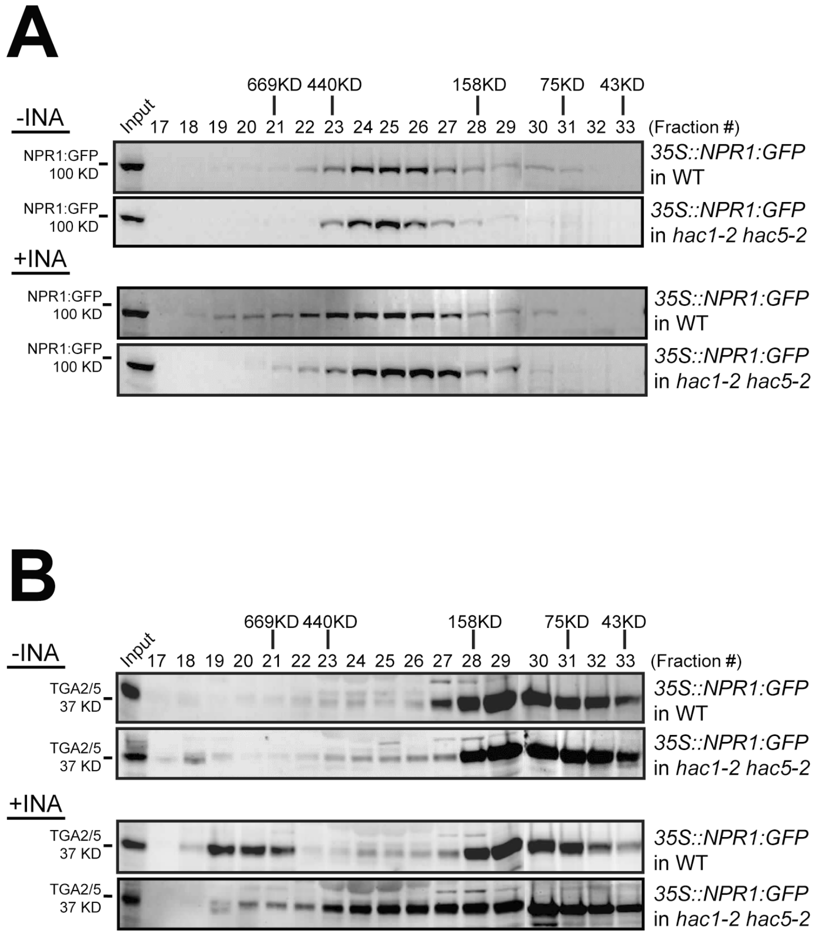

Supplementary Figure S12. Fractionation of the HAC-NPR1-TGA complex using *35S::NPR1:GFP* transgenic plants in WT or *hac1-2 hac5-2* background. Proteins were fractionated by FPLC and subjected to immunoblot analyses with anti-GFP antibody (**A**) or anti-TGA2/5 antibody (**B**). See Figure 5A-D for experimental details.

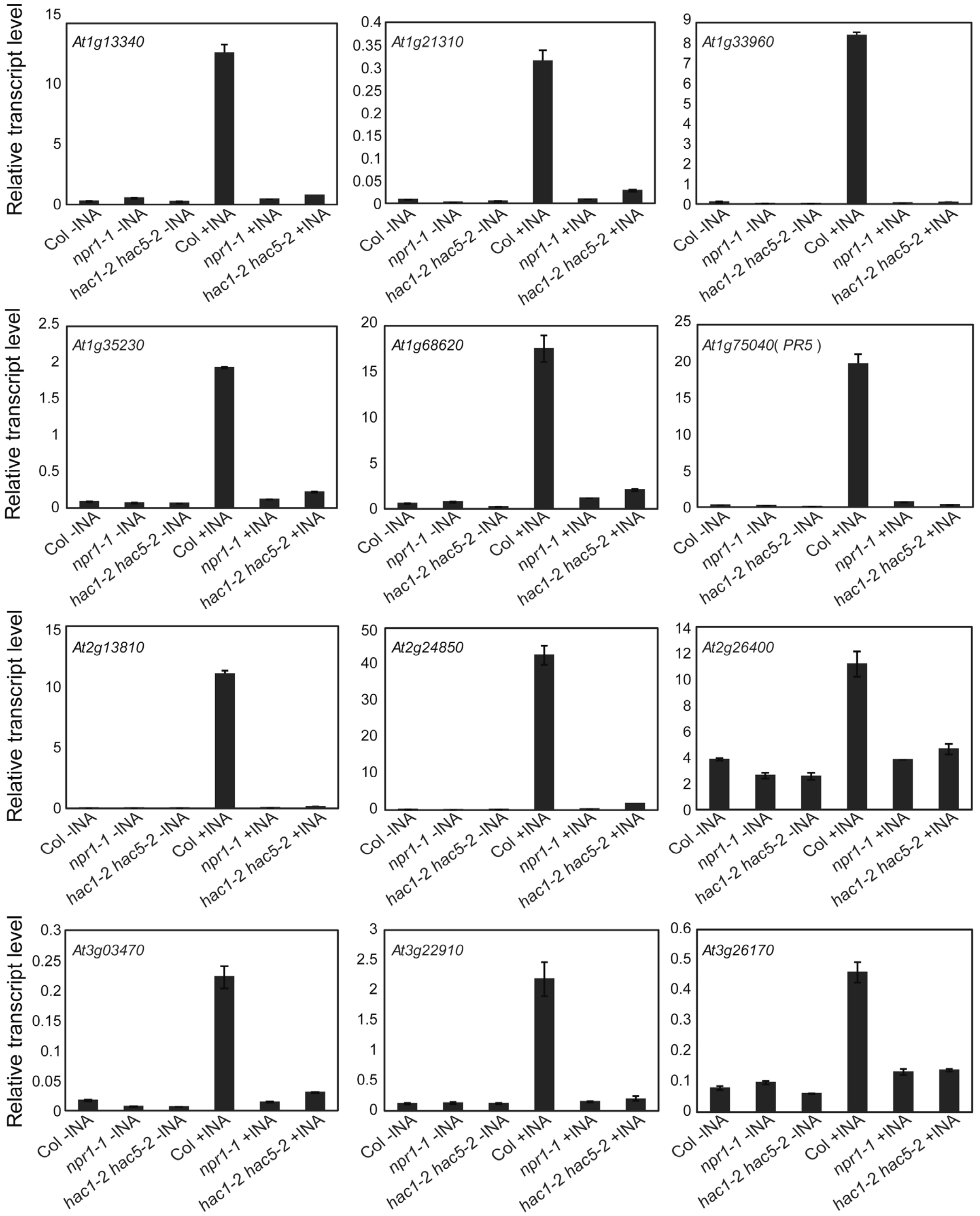
Relative transcript level
At1g13340
At1g21310
At1g33960
At1g35230
At1g68620
At1g75040(PR5)
At2g13810
At2g24850
At2g26400
At3g03470
At3g22910
At3g26170
Col -INA
npr1-1 -INA
hac1-2 hac5-2 -INA
Col +INA
npr1-1 +INA
hac1-2 hac5-2 +INA

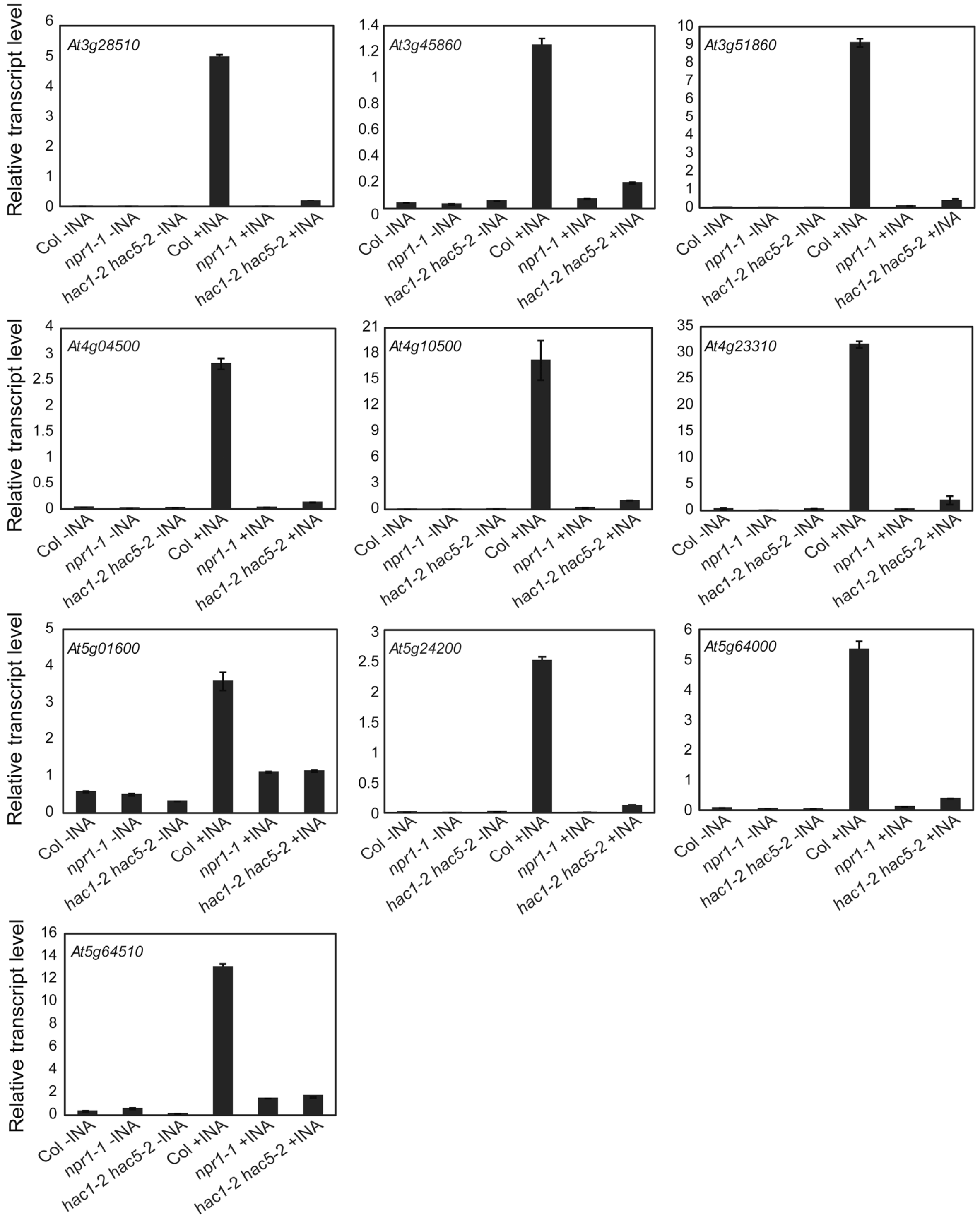

Supplementary Figure S13. RT-qPCR analysis of randomly selected 22 Group 1-gene expression in Col, *npr1-1*, and *hac1-2 hac5-2* treated with INA or not. Means ± SD of duplicates are shown after normalization to *tubulin*.

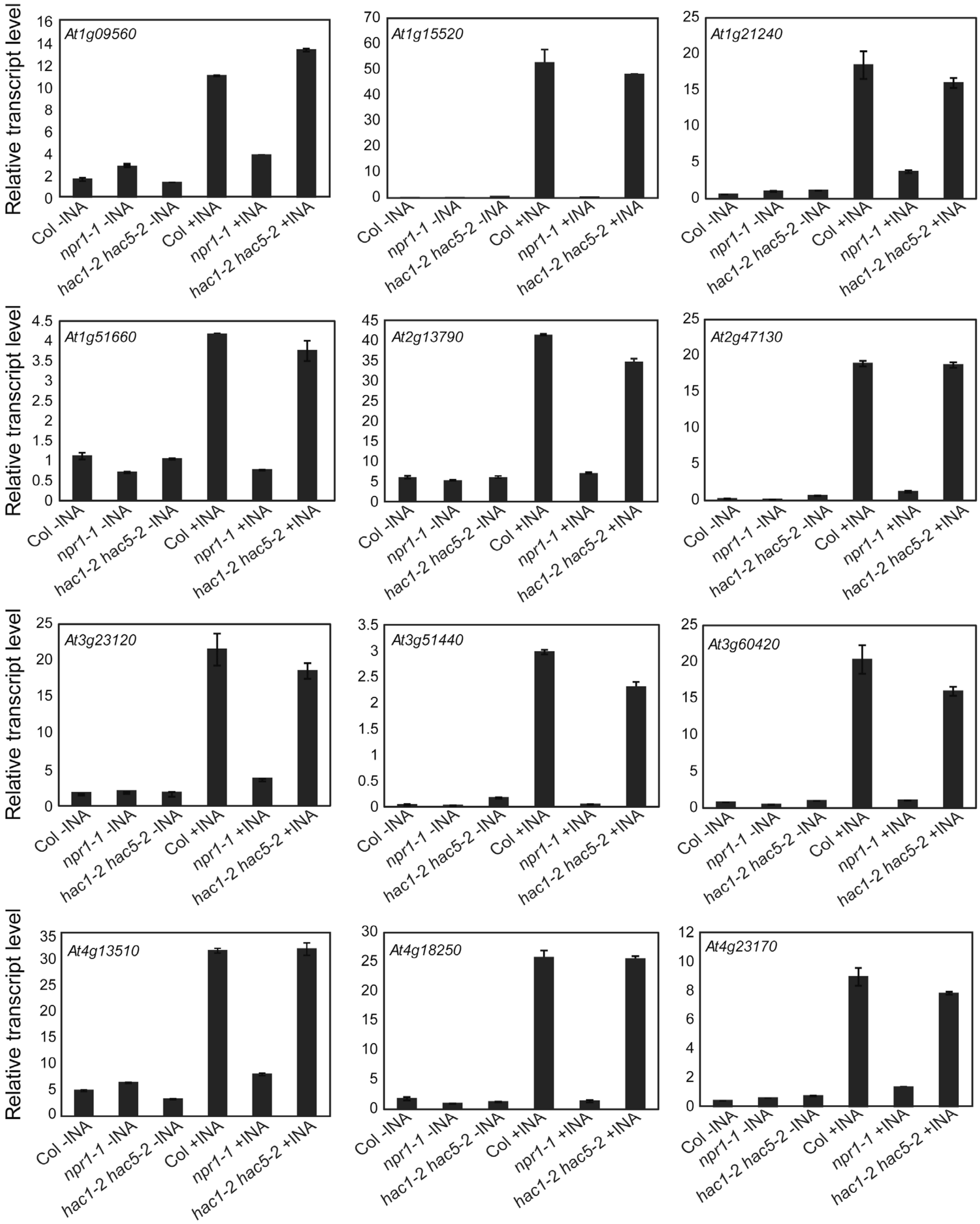
Relative transcript level
At1g09560
At1g15520
At1g21240
At1g51660
At2g13790
At2g47130
At3g23120
At3g51440
At3g60420
At4g13510
At4g18250
At4g23170
Col -INA
npr1-1 -INA
hac1-2 hac5-2 -INA
Col +INA
npr1-1 +INA
hac1-2 hac5-2 +INA

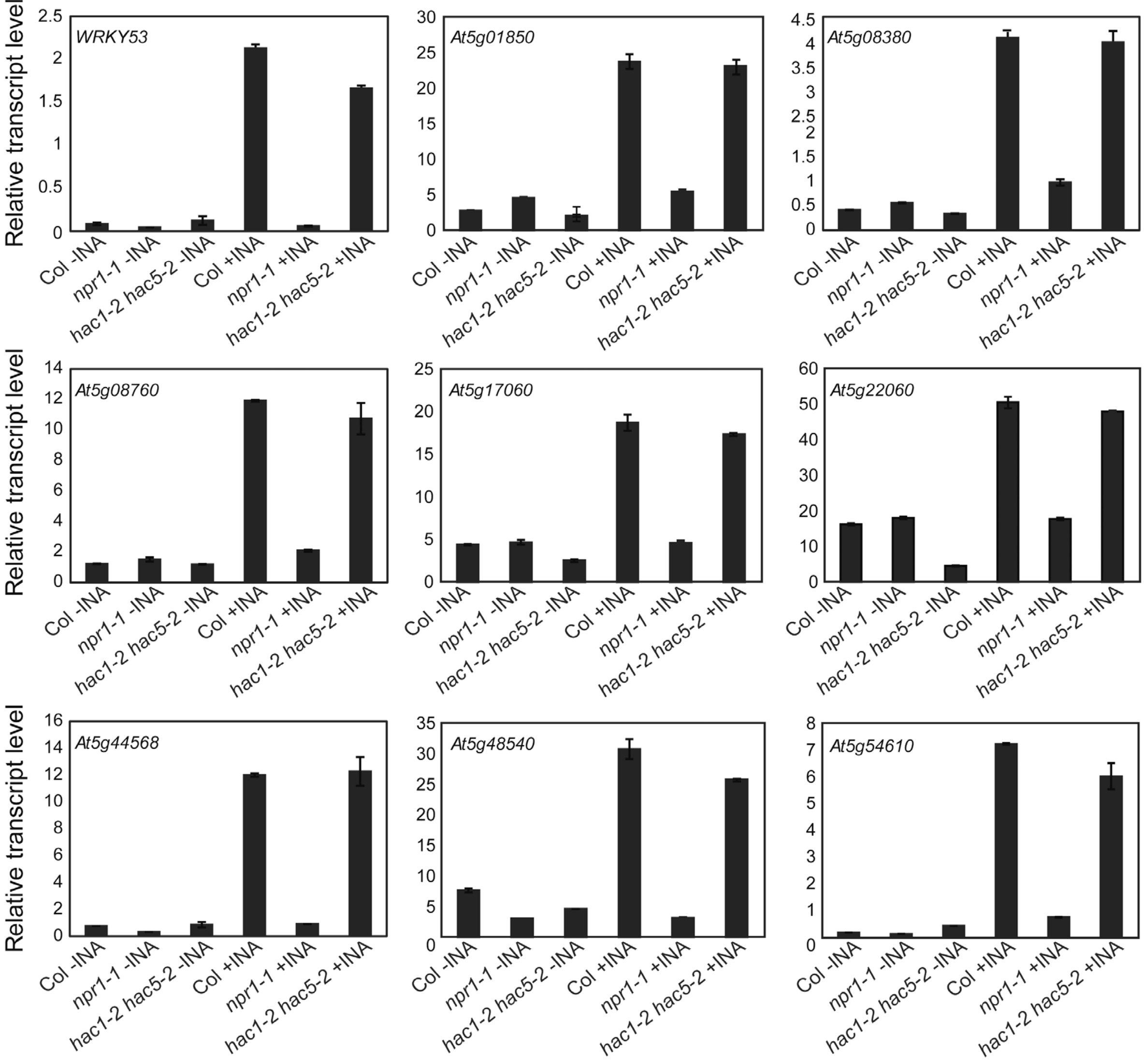

Supplementary Figure S14. RT-qPCR analysis of randomly selected 21 Group 2-gene expression in Col, *npr1-1*, and *hac1-2 hac5-2* treated with INA or not. Means ± SD of duplicates are shown after normalization to *tubulin*.

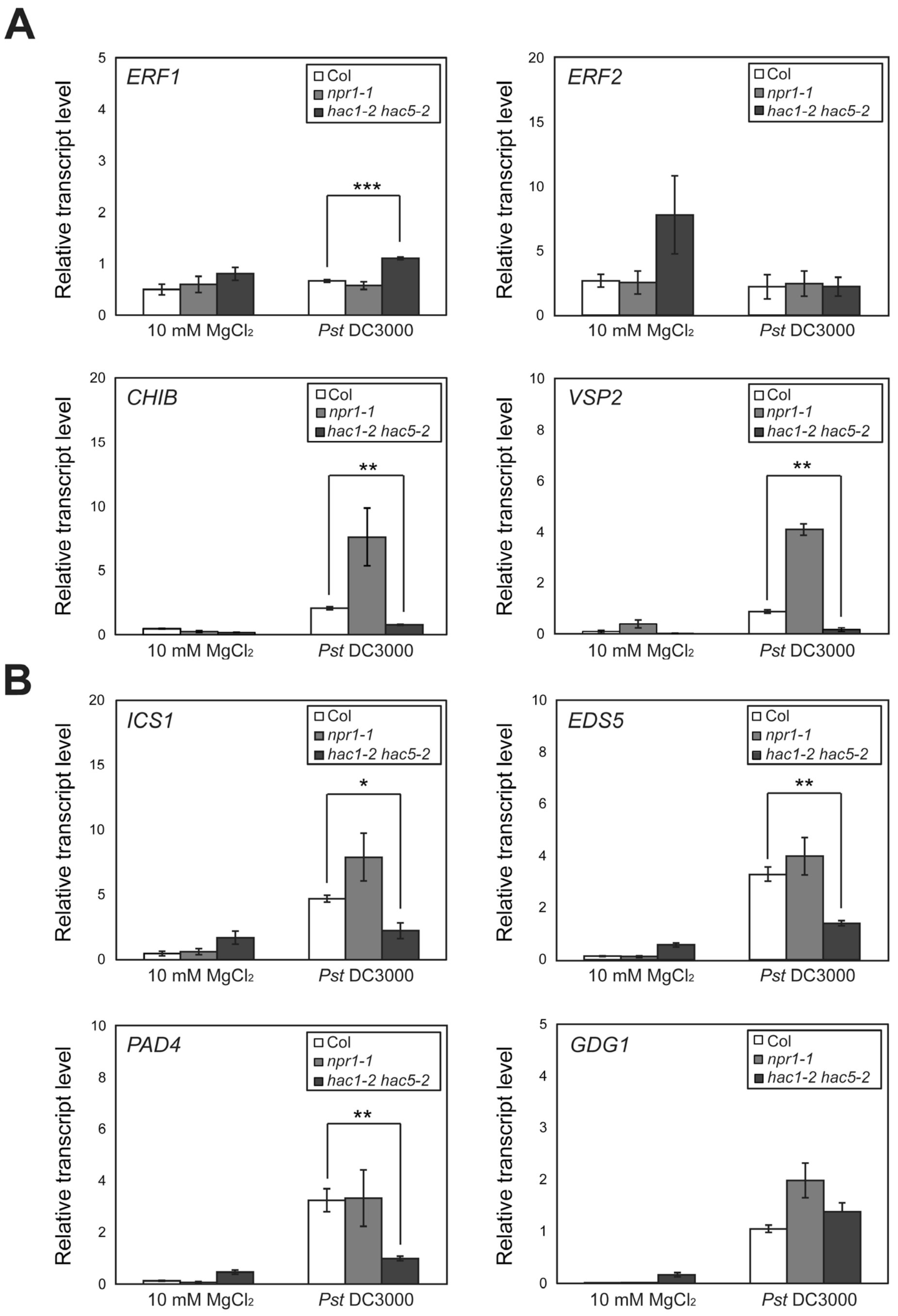

Supplementary Figure S15. RT-qPCR analysis of defense-related gene expression in Col, *npr1-1*, and *hac1-2 hac5-2* plants. (**A**) Expression of the JA/ET response-pathway genes. (**B**) Expression of genes involved in SA biosynthesis or accumulation. 4-w-old plants grown under day-neutral condition (12 hr light/12 hr dark photoperiod) were infiltrated with 10 mM $MgCl_2$ or *Pst* DC3000 at OD_{600} = 0.001 and harvested after 48 hr for RNA extraction. Each gene expression was normalized by *UBQ10*. Means ± SE of three biological replicates are shown. Asterisks indicate statistically significant differences between Col and *hac1-2 hac5-2* (**P* < 0.05, ***P* < 0.01, and ****P* < 0.001 in a Student's *t*-test).

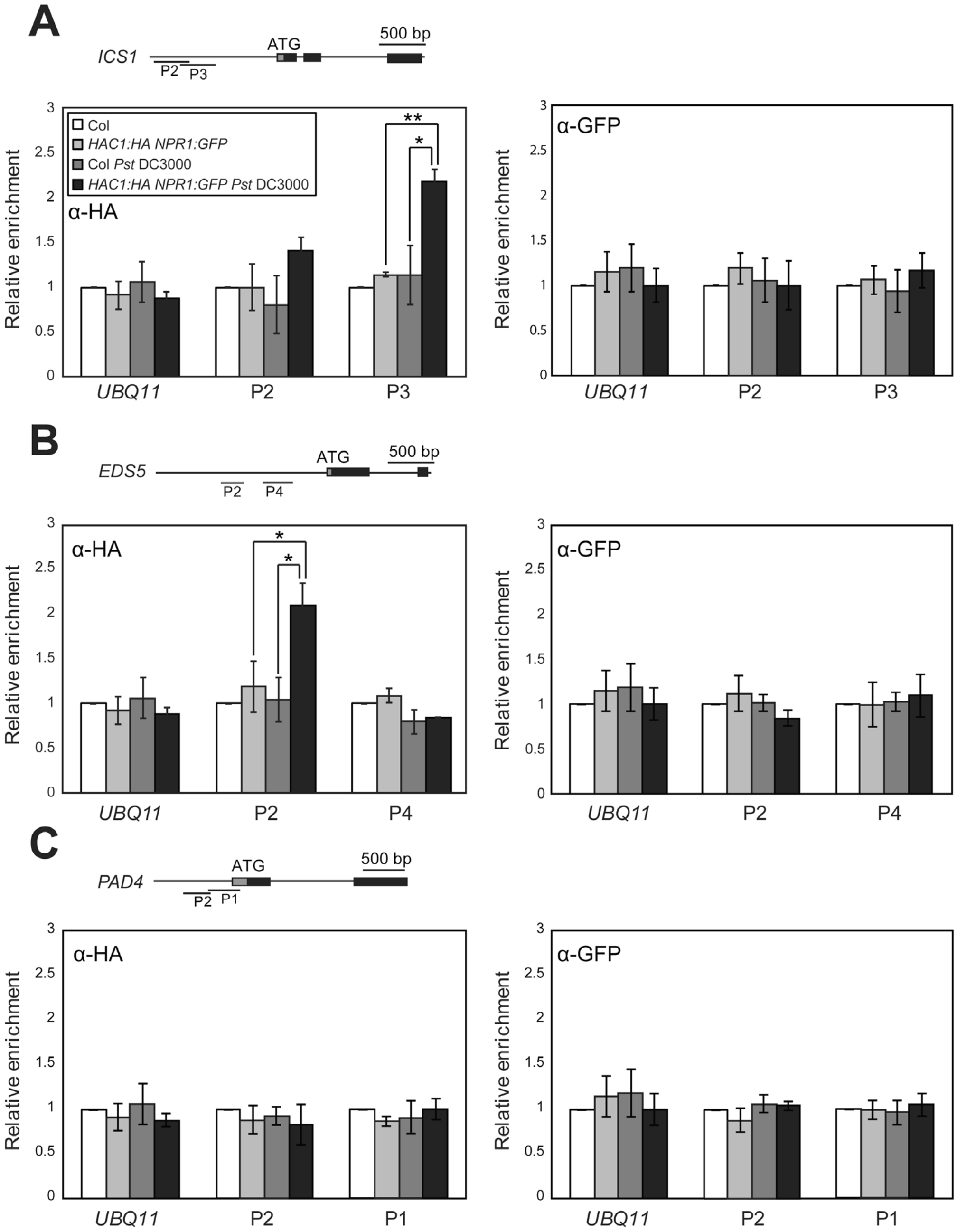

Supplementary Figure S16. HAC1 and NPR1 enrichment within *ICS1*, *EDS5*, and *PAD4* chromatin before and after pathogen infection. (**A** and **B**) ChIP assays showing pathogen-dependent targeting of HAC1:HA but not NPR1:GFP to *ICS1* (**A**) and *EDS5* (**B**) chromatin. (**C**) No targeting activity was detected in the regions tested at *PAD4*. Anti-HA or anti-GFP antibody was used to detect HAC1:HA and NPR1:GFP enrichment, respectively. Asterisks indicate statistically significant differences (**P* < 0.05 and ***P* < 0.01 in a Student's *t*-test). Shown are means ± SE of three independent ChIP experiments performed in triplicates. The uninfected Col levels were set to 1 after normalization by input. See Supplementary Figure S3 for plant sampling.

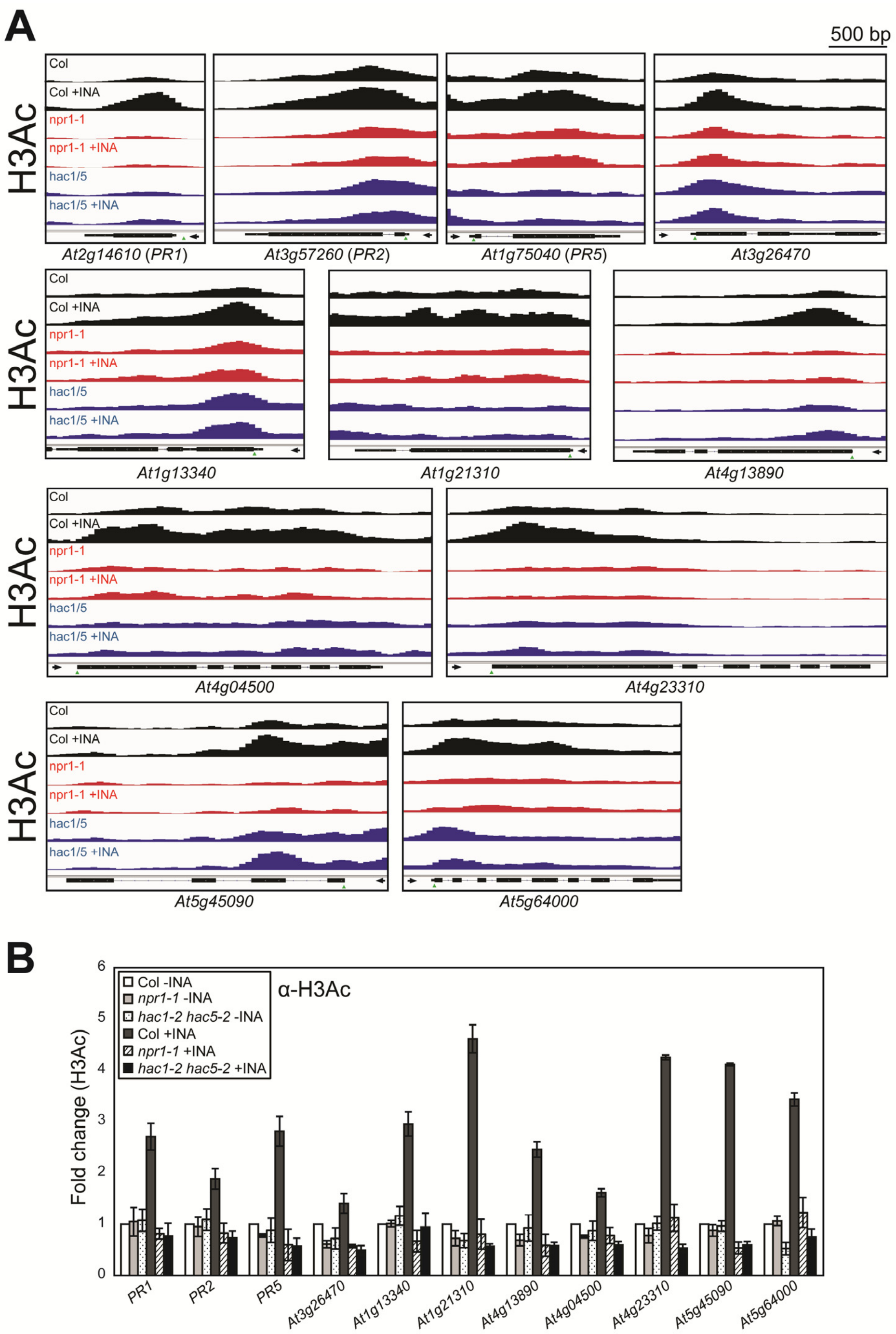

Supplementary Figure S17. Visualization and confirmation of H3Ac ChIP-seq data. (**A**) Integrative Genomics Viewer (IGV) snapshot images of H3Ac ChIP-seq data for 11 selected Group 1-gene loci in Col (black), *npr1-1* (red), and *hac1-2 hac5-2* (blue) plants either treated with INA or not. (**B**) ChIP-qPCR analysis of H3Ac levels for the 11 loci shown in (**A**). The region in each locus tested for ChIP-qPCR is indicated with green triangle within the schematics in (**A**). Means ± SE of triplicates are shown after normalization to input and Col-INA level. See Figure 6 for plant sampling.

Supplementary Table S1. List of all transgenic or multiple-mutant plants used in this study.

Transgenic plant	How to generate
HAC1:HA	Kim et al., 2015 (34)
35S::HAC1:FLAG	By introducing *35S::HAC1:FLAG-DES* into Col
35S::NPR1:GFP	Obtained from X. Dong (Duke University, Durham, North Carolina, USA)
NPR1:GFP in *npr1-1*	By introducing *pNPR1::NPR1:GFP-DES* into *npr1-1*
TGA2:FLAG	By introducing *pTGA2::TGA2:FLAG-DES* into Col
HAC1:HA in *npr1-1*	By crossing *HAC1:HA* with *npr1-1*
HAC1:HA in *tga2 tga5 tga6*	By crossing *HAC1:HA* with *tga2 tga5 tga6*
HAC1:HA 35S::NPR1:GFP	By crossing *HAC1:HA* with *35S::NPR1:GFP* in *npr1-1*
HAC1:HA 35S::NPR1:GFP in *tga2 tga5 tga6*	By crossing *HAC1:HA 35S::NPR1:GFP* with *tga2 tga5 tga6*
HAC1:HA NPR1:GFP	By crossing *HAC1:HA* with *NPR1:GFP* in *npr1-1*
35S::NPR1:GFP in *hac1-2 hac5-2*	By crossing *35S::NPR1:GFP* with *hac1-2* (+/-) *hac5-2* (-/-) and PCR-based genotyping in the following generations
35S::NPR1:GFP TGA2:FLAG in *hac1-2* (+/-) *hac5-2* (-/-)	By crossing *35S::NPR1:GFP* in *hac1-2* (+/-) *hac5-2* (-/-) with *TGA2:FLAG* and PCR-based genotyping in the following generations
35S::NPR1:GFP TGA2:FLAG in *hac1-2* (-/-) *hac5-2* (-/-)	By crossing *35S::NPR1:GFP* in *hac1-2* (+/-) *hac5-2* (-/-) with *TGA2:FLAG* and PCR-based genotyping in the following generations
npr1-1 hac1-2 hac5-2	By crossing *npr1-1* with *hac1-2* (+/-) *hac5-2* (-/-)
tga2 tga5 tga6	By crossing *tga2 tga5* with *tga6*

Supplementary Table S2. Primers used for *HAC1:HA*, *NPR1:GFP*, and *TGA2:FLAG* constructs.

Name	Sequence
HAC1-gate-F	5’-CACCGATTTGGGAAAACCTGAATTCATTCGCT-3’
HAC1-R7	5’-ACCTGAGCCCCCAGCGACTTCTGCAGCTC-3’
NPR1 ORF-F (NdeI)	5’-CACCCATATGGACACCACCAT TGATGGATTCG-3’
NPR1 ORF-R (w/o stop)	5’-CCGACGACGATGAGAGAGTTT-3’
NPR1 P-F (NotI)	5’-CAAGGCGGCCGCGTTACTGTATAGAAAATAGTGTCCC-3’
NPR1 P-R (NdeI)	5’-CTTGCATATGCAACAGGTTCCGATGAATTGAAATTC-3’
TGA2 ORF-F (NdeI)	5’-CACCCATATGGCTGATACCAGTCCGAGAAC-3’
TGA2-R (w/o stop)	5’-CTCTCTGGGTCGAGCAAGCCATAAGG-3’
TGA2 P-F (NotI)	5’-CAAGGCGGCCGCTAATGAGTTAAGAATAGAGAATG-3’
TGA2 P-R (NdeI)	5’-CTTGCATATGATTACTTTCTCACCACTTTTCTGTAC-3’

Supplementary Table S3. Primers used for Yeast-Two-Hybrid constructs.

Name	Sequence
NdeI-NPR1-F	5'-TGCATATGGACACCACCATTGATGGATTCG-3'
BamHI-NPR1-R	5'-CAGGATCCCCGACGACGATGAGAGAGTTT-3'
BamHI-NPR1-ANK-R	5'-AATCCCGGGTCATGCGATCATGAGTGCGGTTCTACC-3'
NdeI-NPR1 Δ370-F	5'-ATTCATATGAAACAAGCCACTATGGCGGTTG-3'
NdeI-NPR1 Δ513-F	5'-ATTCATATGGCAGTGCTCGACCAGATTATG-3'
NPR1-Stop-R	5'-TCACCGACGACGATGAGAGAGTTTACGG-3'
NdeI-HAC1-F	5'-TAACATATGAATGTTCAGGCTCACATGTCGGG-3'
SalI-HAC1-Stop-R	5'-AATGTCGACTTAACCTGAGCCCCCAGCGACTTCTG-3'
SmaI-HAC1-N-F	5'-CGCCCGGGTATGTCGGGGCAGGTTTCAAAC-3'
SalI-HAC1-N-R	5'-GCAGTCGACTTTTGATGTATGTTCAGTAG-3'
NcoI-HAC1-C1-F	5'-GGGCCATGGAGGTGGAGAAAGAACCTGAATCACTT-3'
BamHI-HAC1-C1-R	5'-CCCGGATCCTTTCTTGAGCATTCCTTTCTTATTTCC-3'
NcoI-HAC1-C2-F	5'-GGGCCATGGAGTTTTGTATTCCATGTTATAATGAATCCC-3'
BamHI-HAC1-C2-R	5'-CCCGGATCCGCCTTGACCAGTTTCAATGTCAAG-3'
NcoI-HAC1-C3-F	5'-GGGCCATGGAGACCATTACTAAAAGGGCTCTAAAAG-3'
BamHI-HAC1-C3-R	5'-CCCGGATCCTTAACCTGAGCCCCCAGCGA-3'
NdeI- TAZ^N-F	5'-ATTCATATGAGAAATGGAAATGGCAACCGGGATCCG-3'
TAZ^N-R	5'-TCACTGTTGCTGTAGGTAGGCCTTCACAGG-3'
NdeI- TAZ^C-F	5'-ATTCATATGGCTCAAAACAAAGAAGCGAGGCAATTGC-3'
TAZ^C-R	5'-TCATCTCAGATGCTCCTTTAGGTCCCTGC-3'

Supplementary Table S4. Primers used for RT-qPCR analyses.

Gene	Name	Sequence
UBQ10	qUBQ-F	5’-GGCCTTGTATAATCCCTGATGAATAAG-3’
	qUBQ-R	5’-AAAGAGATAACAGGAACGGAAACATAGT-3’
Tubulin	TUB-F	5’-GTGGTAGTGAAGAATCAAGAGCACC-3’
	TUB-R	5’-GAACCCTAAAGTTCTCAGGCTCCAC-3’
PR1	qPR1-F	5’-GCCGTGAACATGTGGGTTAG-3’
	qPR1-R	5’-GGCACATCCGAGTCTCACTG-3’
PR2	qPR2-F	5’-GATCGTTGGAAATCGTGGTG-3’
	qPR2-R	5’-TAGCTTTCCCTGGCCTTCTC-3’
AT1G13340	F	5’-CTCAAGCCATCTCTGATGTCAC-3’
	R	5’-TCGAGAGTGTTTTGGTCTTTGA-3’
AT1G21310	F	5’-GTCTCCAATGGCCTCTTTAGTG-3’
	R	5’-GGTGGTGGAGGAGAAGAATAGA-3’
AT1G33960	F	5’-TTATCGACTTGGTCAGAAAGCA-3’
	R	5’-TTCTGAATGCCCTTTTGATTCT-3’
AT1G35230	F	5’-TACTGAATCTCCACCAGCTCCT-3’
	R	5’-ACGAGGGAGACTCTGCTAACTG-3’
AT1G68620	F	5’-ATGGACCAGTCGTAGACGAAGT-3’
	R	5’-GGGTAGTGAGGGATCAACACAT-3’
AT1G75040	F	5’-TGCTTAAGGTCATGGATCAGAA-3’
	R	5’-CAAGTTTCCGGCTTATCGTTAG-3’
AT2G13810	F	5’-CTGGTTATGTTGCATCCAGAAA-3’
	R	5’-AACGTGGACTACCATCTTCGAT-3’
AT2G24850	F	5’-GAAAGACCATTTTGTTCCCAAC-3’
	R	5’-TGGGTGCGTAAGAGTTAGCC-3’
AT2G26400	F	5’-ATATGAAGGCAATGCGTCTTTT-3’
	R	5’-TGGACACACCTCACATAAGTCC-3’
AT3G03470	F	5’-AGAAGGAGGGAAGAAGAGGAAA-3’
	R	5’-TCTGGATACTTCACCATGATCG-3’
AT3G22910	F	5’-CCCTCGTTCTCTTTCATACACC-3’
	R	5’-CTAAGCCATTAGGACCACCAAG-3’
AT3G26170	F	5’-GAAAAGATCGAAGAGCTCGTGT-3’
	R	5’-CGTTGAGTCTCTTGTGTTGTCC-3’
AT3G28510	F	5’-TGTGAAGGTGAAGTGGTATTCG-3’
	R	5’-GGTGTCGCCTATGGAAACTAAG-3’
AT3G45860	F	5’-CGCTTCATACTCTACCGGATTC-3’
	R	5’-TTACGGCAAACTTCTTGTGAGA-3’
AT3G51860	F	5’-TTCCATGCAAAACTCTCAAGAA-3’
	R	5’-TTGTAAGAATTGGCAAGAATGG-3’
AT4G04500	F	5’-GTTTGGCAGAGATTCCAAAAAC-3’
	R	5’-TGTGTCTTCAATCACATGTTCG-3’
AT4G10500	F	5’-ACTCCATTGCTTTCCCATAGAA-3’

	R	5’-CTCTGAGATGGCCTCAAGAAGT-3’
AT4G23310	F	5’-GCCTCCTCTAGCTACTCCAGTG-3’
	R	5’-CACAGTTATGGCAAACTTCTGG-3’
AT5G01600	F	5’-CTCCTAAGCCACTACTCCCTCA-3’
	R	5’-ATGTTGTTTGTGTCCACCGTAG-3’
AT5G24200	F	5’-CCCAACTCGATAGTACCTCCAC-3’
	R	5’-GAAGGCGATACGAATGTTAAGC-3’
AT5G64000	F	5’-GGAGCTCCAACCTGATAAACTG-3’
	R	5’-GAGAGCTGGTGTACGATTCCTC-3’
AT5G64510	F	5’-GAAGTCAAGGTTTCTGGGTTTG-3’
	R	5’-GTATTCCCATCGGTTCACATCT-3’
AT1G09560	F	5’-TTCCAGAAGAACAATGGTGATG-3’
	R	5’-CAAGATGTTGTCAGGAACAGGA-3’
AT1G15520	F	5’-TTGATCGTCTCAGGAAAGGAAT-3’
	R	5’-CCATTTGATGAGCCTCTCTAGC-3’
AT1G21240	F	5’-GAAGTCCCCTTGTTGGTCTATG-3’
	R	5’-AGAGTTCCAGCGACTTCTATCG-3’
AT1G51660	F	5’-CCTCTTCCTCTCCCACCTACTT-3’
	R	5’-TCCGATACGGTTACCTCTCACT-3’
AT2G13790	F	5’-TGAAGAAGACCCAGAGGTTCAT-3’
	R	5’-CCAAAACCACCTCTACCCAATA-3’
AT2G47130	F	5’-AGCTTTCTCGACTTGAATCTGG-3’
	R	5’-GCTGGTCGTACATACGATTGAC-3’
AT3G23120	F	5’-GCCAACTTAACCAAGCTTTCTC-3’
	R	5’-GGATTTGAAGTGATTGGAGGAG-3’
AT3G51440	F	5’-CACTCTCTACCAACTCGACACG-3’
	R	5’-ATTGAGAAGACCGACTCCGATA-3’
AT3G60420	F	5’-GGCCTTTAGAACTGGTCAGAGA-3’
	R	5’-GACATAGCAATGGGATCGAAAT-3’
AT4G13510	F	5’-GCCTCTGCTGACTACTCCAACT-3’
	R	5’-AAACCCGGTTAAGAAAGAGGAA-3’
AT4G18250	F	5’-CTATGCTCCATCGACTCAACAG-3’
	R	5’-ACCGTACTCAACACTGATGGTG-3’
AT2G23170	F	5’-GGGATCAACTTGAAACCAATGT-3’
	R	5’-TAGCTCCACAAGTTCGGATTTT-3’
AT4G23810	F	5’-AGATGTTACCAAAGTGGTCAGAAA-3’
	R	5’-TAACTCCTTGGGAATTTGGCGCCT-3’
AT5G01850	F	5’-TACCAAGGAAGGTATGGTCGTC-3’
	R	5’-ATATTGACCTCACGGACGAAAC-3’
AT5G08380	F	5’-GGTCGAGTCTTCGAGATCAGTT-3’
	R	5’-ATGTTGCAGCTAAAATGGTTCC-3’
AT5G08760	F	5’-AGGTACTCGGATTCTCCTCGAT-3’
	R	5’-GCCTTTTCCTTTGGTTGAGTTA-3’

AT5G17060	F	5’-GTGGTGGATTCCTTAGATCGAG-3’
	R	5’-CTCTCATGTCCTGTTTGTTTGC-3’
AT5G22060	F	5’-ATGATCAATATGGGGAAGATGC-3’
	R	5’-CCACCACTACCAAAGAAGGAAG-3’
AT5G44568	F	5’-AACAAAGGAACAAATTGCGTTT-3’
	R	5’-CCTTTATCTTTAGGCGATGCAG-3’
AT5G48540	F	5’-GACGCTTGTCTCAAAGACTCCT-3’
	R	5’-TCCTCTACATTGAGCAAGTCCA-3’
AT5G54610	F	5’-CTCAATCGGGTAGTGTTGATGA-3’
	R	5’-TGGAGAGGTGTGTGGATGATAG-3’
ERF1	qRT F	5’-ATTCTTTCTCATCCTCTTCTTCT-3’
	qRT R	5’-CGAATCTCTTATCTCCGCCG-3’
ERF2	qRT F	5’-GGTTTGGTTAGGGACGTTTG-3’
	qRT R	5’-TCAACTTCCCGTTTTCAGACGA-3’
VSP2	qRT F	5’-TTCTATGCCAAAGGACTTGC-3’
	qRT R	5’-GGAGTCCAGGGGTTGATGCT-3’
CHIB	qRT F	5’-GGATGACTGCTCAGCCTCCC-3’
	qRT R	5’-TCCACCGTTAATGATGTTCG-3’
ICS1	qRT F	5’-CATTGATCTATGCGGGGACA-3’
	qRT R	5’-TGGACAAAAGCTCGTACCTGA-3’
EDS5	qRT F	5’-CCACTCTCTCAAACGGCTCA-3’
	qRT R	5’-ACCGCTGTTCCGATAACTCC-3’
PAD4	qRT F	5’-GCAAGCAAAAGAGTGGTTGG-3’
	qRT R	5’-TCGTCACCAATGTATTCGCA-3’
GDG1	qRT F	5’-CAAATTTCGCTGGCTTGTATAGG-3’
	qRT R	5’-CCTCTCCCTCTTTGGTATCTAC-3’

Supplementary Table S5. Primers used for ChIP assays.

Locus	Name	Sequence
UBQ11	ChIP-F	5’-TCAGTATATGTCTCGCAGCAAACTATC-3’
	ChIP-R	5’-GACGACTCGGTCGGTCACG-3’
ACTIN2	ChIP-F	5’-GATCCGTTCGCTTGATTTTGC-3’
	ChIP-F	5’-ACAAGCACGGATCGAATCACA-3’
PR1-P2	P2-F	5’-ATGGGTGATCTATTGACTGTTT-3’
	P6-R	5’-ATCACTCTTGCCTATGGCTG-3’
PR1-P3	P3-F	5’-GCCAAACTGTCCGATACGATT-3’
	P7-R	5’-TGTCATTCAGTTGTTTTGTGTTTTT-3’
PR1-P4	P8-F	5’-ACGTGAGATCTATAGTTAAC-3’
	P5-R	5’- CGATTAAAAATCGAGAATAGCCAG-3’
PR1-P5	QRT-F	5’- GCCGTGAACATGTGGGTTAG-3’
	QRT-R	5’- GGCACATCCGAGTCTCACTG-3’
PR2-P1	P4-F	5’-CCCCAGGCTTGGCTCTATAA-3’
	P1-R	5’-GGCTAAGCTCCTTGATTCAGAC-3’
PR2-P2	P5-F	5’-GACGTACGATTAACGGCCAA-3’
	P5-R	5’-TATGATGAATCGCCCAAACC-3’
PR2-P3	P6-F	5’-ATCATGAAGGGGGAAAACGA-3’
	P6-R	5’-TTGGCTTGTGGGTCTAAGGA-3’
ICS1-P2	P2-F	5’-GCCTAAGTGGGTTTCCTAGCA-3’
	P2-R	5’-GTGGATTCTCTTTCTAAGCGG-3’
ICS1-P3	P3-F	5’-AAAGTGCAAACCGCTTCCGT-3’
	P3-R	5’-CATGAATCATTTACTTGGTC-3’
EDS5-P2	P2-F	5’-GGTGTGTTTCGGTGACTTTTG-3’
	P2-R	5’-CACTTGTGAAAATCTTTTTGTCC-3’
EDS5-P4	P4-F	5’-CCTTTTGGGAAAATTGTCGTC-3’
	P4-R	5’-GAGACAATGAAAAAGACTTCTATTG-3’
PAD4-P1	P1-F	5’-CCATTCCGATTACATAGGAT-3’
	P1-R	5’-GTAGAGAGTTGCAGAACGATGATG-3’
PAD4-P2	P2-F	5’-TAGTAGAGGTAGACCACAAC-3’
	P2-R	5’-TTAATCATTGTGTGTAGAGG-3’

AT1G13340	F	5’-CGTAACGCGTCTTCTCCTCT-3’
	R	5’-TGAGAGAGTCTGGCTTGACG-3’
AT1G21310	F	5’-TGAGAGTCGGAGAGACCAACA-3’
	R	5’-TTGCCACTAAAGAGGCCATT-3’
AT1G75040	F	5’-GCA AAG AAA ATT CAG AGA ACC AA-3’
	R	5’-TTGTGATGAACACGAGGAAGA-3’
AT3G26470	F	5’-TATGGAGATGTCACTTGTCACTTC-3’
	R	5’-TCTCACCGGCGAAATAATCAGTG-3’
AT4G04500	F	5’-TGCTACCACACCTAGTCAAGTCA-3’
	R	5’-AAAAGTCGCCGTAGCAAATG-3’
AT4G13890	F	5’-TGGAATAACTTGCTAAAGGCATCA-3’
	R	5’-GACGAAGTCAAGATGCGTGTTTCCC-3’
AT4G23310	F	5’-TTCCACAGTGCAACAAGACC-3’
	R	5’-TGGGACAGCTGTGGTATCTG-3’
AT5G45090	F	5’-GATTGAGAGATTAAGAGAGAGCTTT-3’
	R	5’-CCTCCGCCTTCTTGTTTCTCCAGT-3’
AT5G64000	F	5’-AGATGGTTTGCACCAACTCC-3’
	R	5’-TTGTTACCTGGCTGAGACGA-3’
