# Supplementary material for: Salicylic acid-induced transcriptional reprogramming by the HAC–NPR1–TGA histone acetyltransferase complex in Arabidopsis
Source: Nucleic Acids Res. 2018 Sep 17;46(22):11712–25. doi: 10.1093/nar/gky847 (PMC6294559; doi:10.1093/nar/gky847)
Supplement: Supplementary Data [file gky847_supplemental_files.zip › Supplementary Data.pdf]

SUPPLEMENTARY DATA

Supplementary Figure S1. HAC1/5 regulate pathogen-induced *PR2* transcription and histone acetylation.

Supplementary Figure S2. Direct targeting of HAC1, NPR1, and TGA2 to *PR*2 chromatin.

Supplementary Figure S3. HAC1 and NPR1 are targeted to the *PR1* and *PR*2 loci in a pathogen-dependent manner.

Supplementary Figure S4. NPR1 enrichment within *PR1* chromatin in *35S::NPR1:GFP npr1-1* plants either treated with INA or not.

Supplementary Figure S5. TGA2:FLAG forms a complex with NPR1:GFP *in vivo*.

Supplementary Figure S6. Adult phenotypes of Col, *npr1-1*, *hac1-2 hac5-2,* and *npr1-1 hac1-2 hac5-2* mutant plants.

Supplementary Figure S7. Subcellular localization of HAC1, NPR1, and TGA2/5.

Supplementary Figure S8. *In vivo* interactions among HAC1, NPR1, and TGA2/5.

Supplementary Figure S9. *In vivo* interactions among HAC1, NPR1, and TGA2/5 in Col, *HAC1:HA NPR1:GFP*, and *HAC1:HA npr1-1* plants without or with pathogen infection.

Supplementary Figure S10. Interaction between HAC1 and NPR1 in yeast.

Supplementary Figure S11. Role of HAC1/5 in TGA2 and NPR1 targeting to *PR1* or *PR2* chromatin.

Supplementary Figure S12. Fractionation of the HAC-NPR1-TGA complex using *35S::NPR1:GFP* transgenic plants in WT or *hac1-2 hac5-2* background.

Supplementary Figure S13. RT-qPCR analysis of randomly selected 22 Group 1-gene expression in Col, *npr1-1*, and *hac1-2 hac5-2* treated with INA or not.

Supplementary Figure S14. RT-qPCR analysis of randomly selected 21 Group 2-gene expression in Col, *npr1-1*, and *hac1-2 hac5-2* treated with INA or not.

Supplementary Figure S15. RT-qPCR analysis of defense-related gene expression in Col, *npr1-1*, and *hac1-2 hac5-2* plants.

Supplementary Figure S16. HAC1 and NPR1 enrichment within *ICS1*, *EDS5*, and *PAD4* chromatin before and after pathogen infection.

Supplementary Figure S17. Visualization and confirmation of H3Ac ChIP-seq data.

Supplementary Table S1. List of all transgenic or multiple-mutant plants used in this study.

Supplementary Table S2. Primers used for *HAC1:HA*, *NPR1:GFP*, and *TGA2:FLAG* constructs.

Supplementary Table S3. Primers used for Yeast-Two-Hybrid constructs.

Supplementary Table S4. Primers used for RT-qPCR analyses.

Supplementary Table S5. Primers used for ChIP assays.

Supplementary Dataset S1. List of genes showing differential expression as identified by RNA seq.

Supplementary Dataset S2. Lists of the Group 1 and Group 2 genes.

Supplementary Dataset S3. H3Ac peak calling by using MACS2.

Supplementary Dataset S4. Differential peaks identified by using MACS2 bdgdiff.

Supplementary Dataset S5. Annotation of differential peaks by using PAVIS.

Supplementary Dataset S6. List of the further selected Group 1 genes.
